# Specific Requirement of the p84/p110γ Complex of PI3Kγ for Antibody‐Activated, Inducible Cross‐Presentation in Murine Type 2 DCs

**DOI:** 10.1002/advs.202401179

**Published:** 2024-10-09

**Authors:** Despoina Koumantou, Aimé Cézaire Adiko, Pierre Bourdely, Mathilde Nugue, Erwan Boedec, Jamel El‐Benna, Renato Monteiro, Cosmin Saveanu, Muriel Laffargue, Matthias P. Wymann, Marc Dalod, Pierre Guermonprez, Loredana Saveanu

**Affiliations:** ^1^ Centre de Recherche sur l'Inflammation INSERM UMR1149 CNRS EMR8252 Faculté de Médecine site Bichat Université Paris Cité Paris 75018 France; ^2^ Laboratoire d'Excellence Inflamex Université Paris Cité Paris 75018 France; ^3^ CNRS INSERM Institut Cochin Paris 75014 France; ^4^ Institut Pasteur RNA Biology of Fungal Pathogens Université Paris Cité Paris 75015 France; ^5^ INSERM UMR 1048, I2MC Toulouse 4 31432 France; ^6^ Department of Biomedicine University of Basel Mattenstrasse 28 Basel CH‐4058 Switzerland; ^7^ CNRS INSERM CIML Centre d'Immunologie de Marseille‐Luminy Turing Center for Living Systems Aix‐Marseille University Marseille 13007 France; ^8^ “Dendritic cells and adaptive immunity” Immunology department Pasteur Institute Paris 75015 France; ^9^ CNRS UMR3738, Département Biologie du Développement et Cellules Souches Institut Pasteur, Université Paris Cité 25‐28 rue du Docteur Roux Paris 75015 France

**Keywords:** PI3K, antigen cross‐presentation, dendritic cells, immune complexes

## Abstract

Cross‐presentation by MHCI is optimally efficient in type 1 dendritic cells (DC) due to their high capacity for antigen processing. However, through specific pathways, other DCs, such as type 2 DCs and inflammatory DCs (iDCs) can also cross‐present antigens. FcγR‐mediated uptake by type 2 DC and iDC subsets mediates antibody‐dependent cross‐presentation and activation of CD8^+^ T cell responses. Here, an important role for the p84 regulatory subunit of PI3Kγ in mediating efficient cross‐presentation of exogenous antigens in otherwise inefficient cross‐presenting cells, such as type 2 DCs and GM‐CSF‐derived iDCs is identified. FcγR‐mediated cross‐presentation is shown in type 2 and iDCs depend on the enzymatic activity of the p84/p110γ complex of PI3Kγ, which controls the activity of the NADPH oxidase NOX2 and ROS production in murine spleen type 2 DCs and GM‐CSF‐derived iDCs. In contrast, p84/p110γ is largely dispensable for cross‐presentation by type 1 DCs. These findings suggest that PI3Kγ‐targeted therapies, currently considered for oncological practice, may interfere with the ability of type 2 DCs and iDCs to cross‐present antigens contained in immune complexes.

## Graphical abstract

Type 2 DCs and inflammatory DCs can cross‐present antibody‐bound antigens. Type 1 DCs, considered the dominant cross‐presenting population, are not involved in this process. In type 2 DCs, antibody‐antigen complexes recognized by FcγR induce the selective activation of the p84/p110 complex of PI3Kγ. This activation promotes antigen cross‐presentation by inducing NOX2‐mediated ROS production in the endocytic pathway.

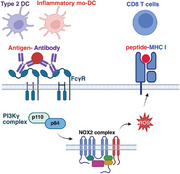

## 1 Introduction

Cross‐presentation is the pathway by which exogenous antigens are processed and presented by professional antigen‐presenting cells (pAPCs), via the class I Major Histocompatibility Complex (MHC‐I), to T cells. This type of MHC‐I antigen presentation is essential for the priming of cytotoxic T cells specific for viruses that do not infect pAPCs, intracellular bacteria, and tumors.^[^ ^1^, ^2^ ^]^

The most efficient cross‐presenting pAPCs are the dendritic cells (DCs). Both human and murine DCs contain in steady state 2 main subsets, the plasmacytoid DCs (pDCs) and the conventional DCs (cDCs). cDCs include lymphoid organ resident DCs and migratory DCs. Based on their phenotype, function, and transcription factor dependency, cDCs are subdivided into type 1 and type 2 cDCs.^[^ ^3^ ^]^ In addition to these steady‐state DC subsets, in inflammatory conditions an important number of DC‐like cells differentiate from classical monocytes,^[^ ^4^, ^5^ ^]^ atypical CD209a monocytes,^[^ ^6^ ^]^ or LyC^+^ monocyte‐DC‐progenitors.^[^ ^7^ ^]^ Not all subsets of DCs are capable of efficient cross‐presentation. In steady‐state conditions, murine type 1 DCs are the main DC subset that internalizes and cross‐presents cell‐associated antigens efficiently. The efficiency of type 1 DCs in cross‐presentation is afforded by a strong ability to uptake cell debris,^[^ ^8^, ^9^ ^]^ dedicated receptors signaling for cross‐presentation,^[^ ^10^ ^]^ and DC1‐associated intracellular machinery that control antigen processing and presentation.^[^ ^11^, ^12^, ^13^ ^]^ Nevertheless, if the antigen is targeted to specific endocytic receptors^[^ ^5^ ^]^ or DCs undergo specific innate activation,^[^ ^14^ ^]^ all DCs subsets are able to cross‐present it to some extent. The transcriptional program that enables monocytes to differentiate into antigen cross‐presenting cells is known,^[^ ^15^ ^]^ but the mechanisms that control antigen processing and presentation remain elusive, and it is unclear whether specific machinery controls inducible cross‐presentation in type 2 DCs and GMCSF‐derived inflammatory DCs.

Important endocytic receptors expressed at high levels by inflammatory cells and at lower levels by type 2 DCs are members of the immunoglobulin Fc Receptor (FcR) family.^[^ ^16^ ^]^ FcγR mediates the uptake of immune complexes (ICs) and of dead cells opsonized by antibodies directed against cell surface proteins.^[^ ^17^ ^]^ Such events occur during viral infections or after the administration of tumor‐targeting monoclonal antibodies (mAbs) used in cancer therapy. For several antitumor mAbs, such as anti‐HER‐2/neu, anti‐MUC1, or anti‐hCD20, there is evidence that in the first step, the mAbs rapidly kill the tumor cells via FcγR‐mediated antibody‐dependent cell cytotoxicity (ADCC), a mechanism by which myeloid cells in mice, and both myeloid and NK cells in humans, recognize and kill antibody‐coated cells. In the second step, mAbs induce a long‐term cytotoxic T‐cell response against the tumor.^[^ ^18^, ^19^, ^20^ ^]^ In a model of FcγR‐humanized mice it has been demonstrated that the rapid ADCC against tumor cells requires hFcγRIIA activation on macrophages, while the long‐term anti‐tumor cytotoxic T cell response requires hFcγRIIA‐mediated cross‐presentation.^[^ ^21^ ^]^ In addition, CD47‐SIRPα inhibition triggers the efficient uptake of cell‐associated antigens in type 2 DCs.^[^ ^22^ ^]^

FcγRs‐mediated cross‐presentation allows type 2 DCs and inflammatory DCs (iDCs) to potently prime naive T cells at low doses of antigen that are not normally cross‐presented by these cells.^[^ ^21^, ^23^ ^]^ The efficiency of FcγRs‐mediated cross‐presentation is not only due to increased antigen uptake via FcγRs endocytosis but also to their downstream Syk‐dependent signaling that induces DC maturation and upregulation of co‐stimulatory molecules.^[^ ^24^ ^]^ Accordingly, FcγR‐mediated cross‐presentation is impaired in the absence of the FcγR ITAM‐bearing γ chain.^[^ ^23^, ^25^ ^]^

Important players in FcγRs signaling are the class I phosphoinositide 3‐kinases (PI3K), which generate phosphatidylinositol (3,4,5)‐trisphosphate (PIP_3_). Class IA PI3Ks include PI3Kα, PI3Kβ, and PI3Kδ, each of which consists of a catalytic subunit (p110α, β or δ) and a regulatory p85‐like subunit, and is activated by Src‐ and Syk family protein kinases. The class IB family has only one unique member, the PI3Kγ.^[^ ^26^ ^]^ Unlike the members of the class IA PI3Ks, PI3Kγ exists as 2 complexes formed by the catalytic subunit p110γ and either a p101 or a p84 regulatory subunit. While PI3Kα and β are ubiquitous enzymes, the expression of PI3Kγ and δ is restricted to immune cells, and their inhibition is often considered a therapeutic approach against lymphoid malignancies, frequently in combination with other treatments, including antitumor mAbs.^[^ ^27^ ^]^

Given the importance of class I PI3K in FcγR signaling^[^ ^24^ ^]^ and following the important role of FcγR‐mediated cross‐presentation in the activation of anti‐tumor cytotoxic T cells,^[^ ^21^ ^]^ we investigated the effect of PI3Kγ function on cross‐presentation of ICs. First, using mice deficient in type 1 DCs, we demonstrated that in vivo type 2 DCs are able to cross‐present antibody‐bound antigens in an FcγR‐dependent manner. Using mice deficient in p110γ, p84, or p101, we also demonstrated that the p110γ‐p84 complex of PI3Kγ, by promoting NOX2 activation and ROS production, enhances the ability of type 2 DCs and GM‐CSF‐differentiated iDCs to cross‐present antigen‐antibody ICs. These results should be taken into consideration when using PI3Kγ inhibitors that target both the p110γ/p84 and p110γ/p101 PI3K complexes, which will affect the ability of type 2 DCs and iDCs to cross‐present IC antigens and produce ROS.

## 2 Results

### 2.1 Antibody‐Bound Antigens can be Efficiently Cross‐Presented in the Absence of Type 1 DCs

FcγRs have been shown to be important for antigen internalization by different subsets of DCs, leading to efficient T‐cell priming. Until now, this has been demonstrated using animal models in which both type 1 and type 2 DCs have been depleted or using FcγRs‐deficient mice in the presence of both types of cDC subsets.^[^ ^17^, ^21^, ^23^, ^28^ ^]^ Thus, the exact role of type 1 or type 2 cDCs in FcγR‐mediated cross‐presentation in vivo is not clearly established despite earlier findings identifying the ability of type 2 DCs to perform this task via an FcγR‐dependent process.^[^ ^23^ ^]^

**Figure 1:**
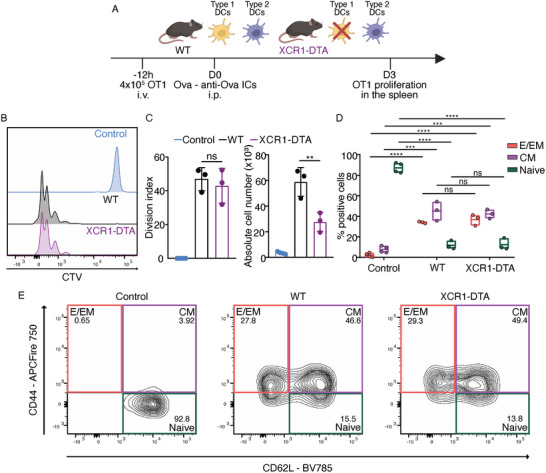
ICs can induce cross‐presentation in vivo in the absence of type 1 DCs. A) Experimental design of the in vivo antigen cross‐presentation assay after OT1 T cell adoptive transfer in WT and XCR1‐DTA mice. 4 × 10^5^ CTV‐labeled OT1 T cells were injected in the recipient mice i.v. and 12 h later the mice received Ova‐anti‐Ova ICs in the peritoneum. OT1 proliferation in the spleen was accessed 3 days after the IC administration by flow cytometry. B) Histograms showing the CTV intensity of proliferating OT1 T cells primed by Ova‐anti‐Ova ICs in WT and XCR1‐DTA mice. Control mice did not receive ICs. OT1 T cells are defined as CD8α and H_2_‐Kb SIINFEKL‐pentamers double positive population. C) Division index (left) and absolute cell number (right) of OT1 T cells in WT and XCR1‐DTA mice after priming by Ova anti‐Ova ICs. Each dot represents an individual mouse (ns: non‐significant; **: 0.001 < *p* < 0.01, one‐way ANOVA test followed by Tukey's multiple comparisons). D) Phenotype of isolated OT1 T cells assessed by the expression of CD62L and CD44 [CD62L^+^: naive, CD44^+^: effector/effector memory (E/EM), CD62L^+^CD44^+^: central memory (CM)]. Minimum, maximum, and mean values are shown in the bars. Each dot represents an individual mouse (ns: non‐significant; ***: 0.0001 < *p* < 0.001; ****: *p *< 0.0001, one‐way ANOVA test followed by Tukey's multiple comparisons). E) Representative contour plots showing the percentage of expression of the CD62L and CD44 surface differentiation markers in the OT1 T cells.

However, type 1 DCs are experts in antigen uptake and could therefore internalize ICs by FcγR‐independent mechanisms such as micropinocytosis,^[^ ^25^ ^]^ making it difficult to assess the selective role of other DC subsets in FcγR‐mediated antigen cross‐presentation. To test whether the antigens bound by the antibodies could be cross‐presented in the absence of type 1 DCs, we used the XCR1‐DTA mice, which are only deficient in the type 1 DC population (Figure S1A, Supporting Information) and compared them with WT mice. The WT or XCR1‐DTA mice were injected with Cell Trace Violet (CTV)‐labeled OT1 T cells carrying the TCR that recognizes the ovalbumin (Ova) SIINFEKL epitope in the context of the H_2_‐Kb allele of murine MHC‐I. Twelve hours later, they received ICs containing the Ova model antigen to induce FcγR‐mediated endocytosis. The proliferation of OT1 T cells was assessed after 72 h by CTV dye dilution in the spleen (**Figure** **1**A). As a negative control for nonspecific T cell proliferation, we used mice that received OT1 T cells but were not injected with ICs. Surprisingly, both WT and XCR1‐DTA mice showed the same ability to induce T cell divisions (Figure 1B,C; Figure S1B, Supporting Information). However, the number of proliferating T cells detected in the XCR1‐DTA mice was lower compared to the WT, suggesting that, as in the case of viral infections, despite the fact that type 2 DCs can prime CD8 T cells, type 1 DCs are still required for optimal T cell proliferation^[^ ^29^, ^30^ ^]^ (Figure 1C).

To assess the differentiation/activation status of the proliferating OT1 T cells, we examined the expression of CD62L and CD44 markers. As expected, almost all OT1 from control mice were naive (CD62L^+^). As in the case of the division index, T cells from both WT and XCR1‐DTA mice showed a similar differentiation pattern, with ≈35% having an effector/effector memory (CD44^+^) phenotype and 42%–45% having a central memory (CD62L^+^ CD44^+^) phenotype (Figure 1D,E).

These results demonstrate that IC can be cross‐presented in vivo in the absence of type 1 DCs. Because FcγR‐mediated cross‐presentation of IC requires the FcγR signaling,^[^ ^31^ ^]^ we decided to investigate the role of class I PI3K, an important component of the FcγR signaling cascade,^[^ ^32^ ^]^ in IC cross‐presentation.

### 2.2 PI3Kγ is not Involved in DC Development of cDCs and GM‐CSF‐Derived iDCs

**Figure 2:**
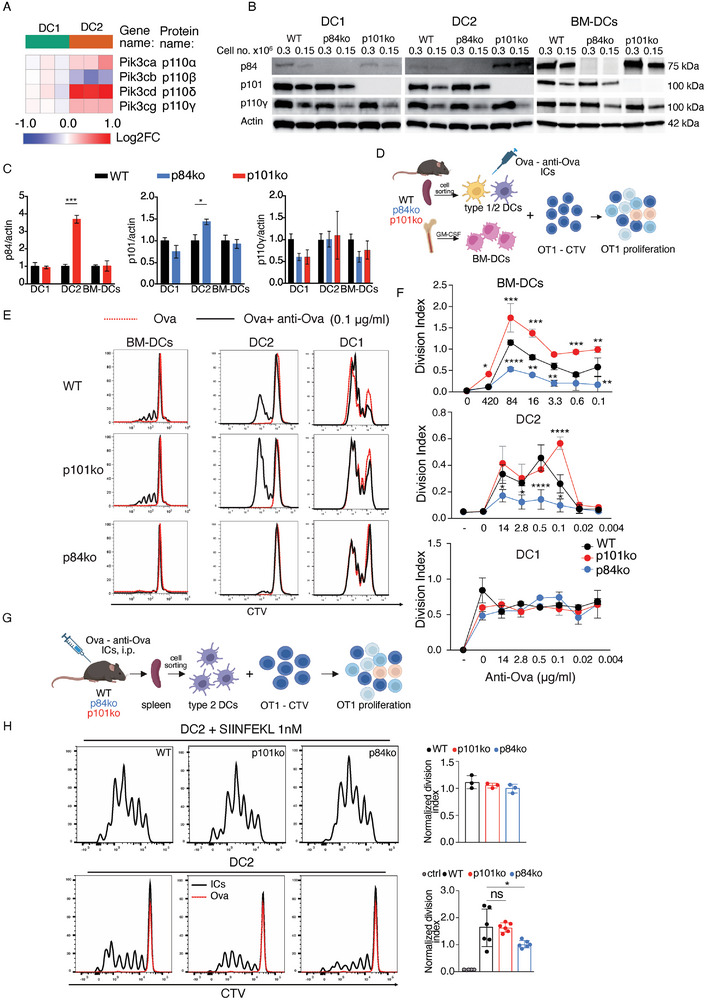
PI3Kγ subunits are expressed in conventional and GM‐CSF BM‐derived iDCs and p84 regulatory subunit controls IC cross‐presentation by type 2 DCs and iDCs. A) Heat map showing the differential expression (Log2 fold change‐Log2FC) of the genes encoding the enzymatically active subunits of all class I PI3Ks in murine splenic conventional DCs, as assessed by bulk RNA sequencing. B) PI3Kγ sub‐unit expression in WT, p84ko, and p101ko conventional DCs and GM‐CSF‐derived iDCs as judged by immunoblotting. Actin was used as a loading control. The blots are representative of 3 independent experiments. C) Quantification of 3 independent immunoblot experiments similar to (B) (*: 0.01 < *p* < 0.05; ***: 0.0001 < *p* < 0.001, unpaired two‐tailed Student *t*‐test). D) Experimental design of the in vitro cross‐presentation assay in WT, p101ko, and p84ko mice. Conventional type 1 and type 2 DCs were sorted from the spleen of Flt3L injected mice and BM‐DCs were differentiated from bone marrow cells using GM‐CSF. The various DC subsets were treated with Ova‐anti‐Ova ICs overnight in different concentrations. The next day they were co‐cultured with CTV‐labeled OT1 T cells, the proliferation of which was assessed after 3 days by flow cytometry. E) In vitro activation of naïve OT1 T cells by WT, p84‐ and p101‐deficient DC subsets cross‐presenting ICs was measured by CTV dye dilution. Shown histograms correspond to an anti‐OVA concentration of 0.1 µg mL^−1^ and are representative of 3 independent experiments realized in duplicate. F) Titrations of anti‐OVA used for OT1 T cell in vitro activation by WT, p84, and p101 deficient GM‐CSF‐derived iDCs, type 2 DCs, and type 1 DCs in the presence of 20 µg mL^−1^ OVA. The division index was calculated according to Angulo et al.^[^ ^72^ ^]^ The graphs show one representative experiment out of 3 (*: 0.01 < *p* < 0.05; **: 0.01<*p* < 0.001***: 0.0001 < *p* < 0.001, two‐way ANOVA test followed by Turkey's multiple comparisons). Each dot represents the mean value of a duplicate. G) Experimental design of the ex vivo cross‐presentation assay. WT, p101ko, and p84 ko mice were injected with Ova‐anti‐Ova ICs, and 3 h later the type 2 DCs were sorted from the spleen and co‐cultured with CTV‐labeled OT1‐T cells in a 1:2 ratio (effector: target). The proliferation of OT1 T cells was measured 3 days later by flow cytometry. H) Ex vivo activation of OT1 T cells by type 2 DCs from WT, p84, and p101‐deficient mice that were injected with IC. Data were derived from 2 independent experiments realized in triplicates. Representative histograms are shown in the figure and the normalized division index against p84ko was plotted in the graphs (ns: non‐significant; *: 0.01 < *p* < 0.05, one‐way ANOVA test followed by Dunnett's multiple comparisons).

Among the class I PI3K family members, PI3Kγ and PI3Kδ catalytic subunits are preferentially expressed in type 2 DCs (**Figure** **2**A). Because PI3Kγ enzymatic activity has been shown to be important for DC‐mediated CD8^+^ T cell priming in viral infections,^[^ ^33^ ^]^ we decided to investigate the function of PI3Kγ and its regulatory subunits in DC subsets and their potential role in FcγR‐mediated cross‐presentation. The p110γ catalytic subunit of PI3Kγ can form complexes with 2 different regulatory subunits, p101 and p84, which are known to have distinct functions in neutrophils.^[^ ^34^ ^]^ Therefore, we focused our study on both p110γ/p101 and p110γ/p84 complexes.

First, we examined their protein expression levels using splenic type 1 and 2 DCs and GM‐CSF‐induced bone marrow‐derived DCs (GM‐CSF‐induced iDCs). Western blot experiments showed that the p101 regulatory subunit and the p110γ catalytic subunit of PI3Kγ are expressed at comparable levels in both types of cDCs and in GM‐CSF‐derived iDCs, whereas p84 expression is significantly higher in GM‐CSF‐derived iDCs than in cDCs (Figure 2B,C). Interestingly, the p84 regulatory subunit was upregulated in type 2 DCs lacking the p101 regulatory subunit, whereas we observed no such regulation in type 1 DCs or GM‐CSF‐derived iDCs (Figure 2B,C).

PI3K is also involved in signaling cascades downstream of the granulocyte and macrophage colony‐stimulating factor (GM‐CSF) and Fms‐like tyrosine kinase 3 ligand (Flt3L) receptors, both of which are essential for the proliferation and survival of iDCs and cDCs respectively. Therefore, we investigated the role of the 2 regulatory subunits of PI3Kγ in the development and survival of different DC subsets. Neither p101 nor p84 were required for the development of splenic cDCs, in basal conditions (Figure S2A,B, Supporting Information). This is consistent with previously published data showing normal development of these cells in p110γ‐deficient mice.^[^ ^33^, ^35^ ^]^ Although highly expressed by GM‐CSF‐derived iDCs, neither the p84 nor the p101 subunit was required for GM‐CSF‐driven differentiation of these cells (Figure S2C,D, Supporting Information). As expected from normal differentiation of p84‐ and p101‐deficient GM‐CSF‐derived iDCs, activation of the key survival pathway PI3K‐mTOR (mammalian target of rapamycin) was not decreased by deletion of p84 or p101 (Figure S2E,F, Supporting Information). We concluded that PI3Kγ is not involved in the differentiation or survival of splenic cDCs and GM‐CSF‐derived iDCs in basal conditions.

### 2.3 The p84 Regulatory Subunit of PI3Kγ Controls Antibody and Fcγ‐Dependent Cross‐Presentation by MHC‐I

To investigate the potential role of p110/p84 and p110/p101 complexes of PI3Kγ in the cross‐presentation of iDCs, we analyzed DCs derived from p84 and p101‐deficient mice. GM‐CSF‐derived iDCs were differentiated from bone marrow precursors of WT, p84, and p101‐deficient mice for 7 days. Splenic DCs were isolated by cell sorting from WT, p84, and p101‐deficient mice as described in Materials and Methods, using the gating strategy shown in Figure S3 (Supporting Information). We performed cDC sorting experiments both under basal conditions and after injection of B16 melanoma cells expressing Flt3L. While under basal conditions the number of cDCs was identical between WT and p84 or p101‐deficient mice (Figure S2A,B, Supporting Information), when we isolated cDCs from mice injected with Flt3L‐expressing B16 melanoma, a small but significant reduction in the percentage of type 1 DCs was observed in mice deficient for p101 or p84 and a slight significant increase in type 2 DCs was observed in mice deficient for p101 (Figure S3, Supporting Information).

Having sufficient numbers of both types of cDC in the absence of PI3Kγ regulatory subunits, we tested their ability to cross‐present antigens in vitro. Cross‐presentation of Ova ICs was measured by activation of OT1 T cells after in vitro uptake of ICs by different DC subsets (Figure 2D). GM‐CSF‐derived iDCs and type 2 DCs cross‐presented low levels of Ova (20 µg mL^−1^) only when the antigen was present in the ICs, whereas type 1 DCs cross‐presented these low levels of antigen even in the absence of anti‐Ova antibodies (Figure 2E,F), consistent with the ability of type 1 DCs to cross‐present ICs in an FcγR‐independent manner.^[^ ^25^ ^]^ In contrast to type 1 DCs, cross‐presentation of ICs was strongly impaired in p84‐deficient GM‐CSF‐derived iDCs and type 2 DCs, whereas it was not affected or even increased in p101‐deficient type 2 DCs and GM‐CSF‐derived iDCs, respectively (Figure 2E,F).

**Figure 3:**
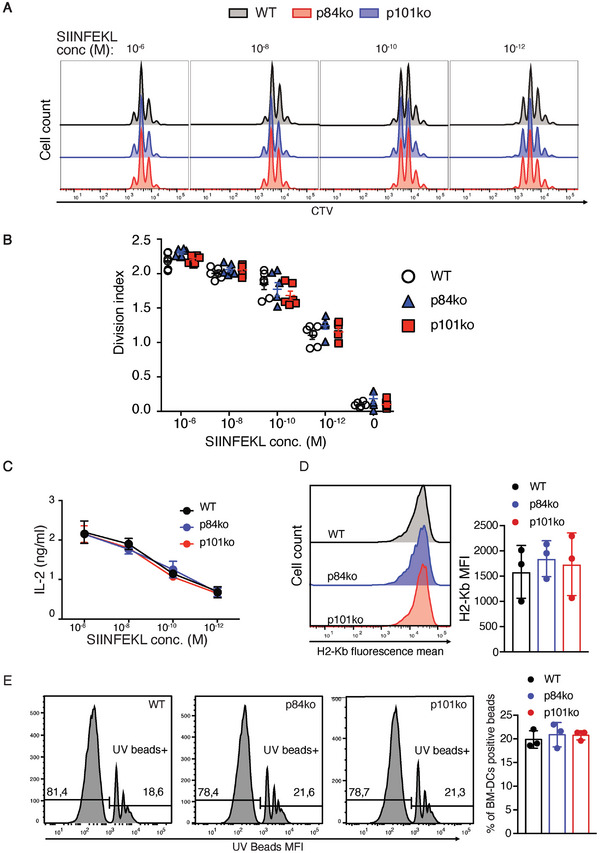
p84 and p101 do not control the presentation of the SIINFEKL peptide to specific CD8^+^ T cells or the antigen uptake. A) WT, p84, and p101‐deficient GM‐CSF‐derived iDCs were pulsed for 2 h with different concentrations of SIINFEKL peptide, washed, and incubated for 3 days with CTV labeled naïve OT1 T cells. Data are representative of 3 independent experiments. B) Graph showing the division index of OT1 T cells after 3 days of co‐culturing with GM‐CSF‐derived iDCs as described in (A). The experiment was performed 3 times in duplicate. C) To have a quantitative evaluation of OT1 T cell activation, the supernatant from the cross‐presentation experiment shown in (A) was used to measure IL‐2 secreted by OT1 T cells by ELISA. Data are representative of 2 independent experiments. D) H_2_‐Kb expression at the cell surface of WT, p84, and p101‐deficient GM‐CSF‐derived iDCs were analyzed by flow cytometry using AF6‐88.5 antibody. The right‐hand graph shows the H_2_‐Kb mean of fluorescence of 3 independent cultures. E) The FcγR‐mediated antigen uptake was investigated by the ability of WT, p84, and p101‐deficient GM‐CSF‐derived iDCs to phagocytose IgG‐coated UV fluorescent beads. The graph shows the percentage of cells that internalized IgG‐coated UV beads. The data are derived from 3 independent experiments.

To investigate whether cross‐presentation of ICs internalized by type 2 DCs also requires the presence of p84 in vivo, we injected Ova‐anti‐Ova ICs into WT or regulatory subunit‐deficient mice, sorted splenic type 2 DCs and co‐cultured them with OT1 T cells (Figure 2G). Similar to the in vitro experiments, p84‐deficient type 2 DCs showed a significantly reduced capacity to induce T cell proliferation (Figure 2H). In addition to antigen processing, the reduced cross‐presentation of ICs by p84‐deficient cells could result from a defect in the expression of MHC‐I or co‐stimulatory molecules, or from a defect in antigen uptake. We tested these alternative hypotheses and found that p84‐deficient DCs expressed similar levels of H_2_‐Kb, and were able to stimulate OT1‐specific T cells as well as their WT counterparts when exogenously pulsed with the SIINFEKL epitope (Figures 2H and **3**A–D) and had a similar ability to internalize antibody‐coated fluorescent beads (Figure 3E).

To take a broader view of the molecules involved in antigen presentation and signaling downstream of Fc receptors, we performed bulk RNA sequencing experiments in splenic type 2 DCs from WT, p84, and p101‐deficient mice. In general, p84 and p101 deletion affected a small number of genes (Figure S4, Supporting Information), indicating that PI3K regulatory subunits do not regulate antigen cross‐presentation at the transcriptional level. Indeed, the expression of genes related to antigen processing and presentation and FcγR signaling molecules (Gene Ontology terms: 0002474 and 1903426), did not show a significant up‐ or down‐regulation (Figure S5A, Supporting Information). In contrast to the transcriptome data, the protein level of activating FcγRs, which are expressed at relatively low levels in cDCs (Figure S6A, Supporting Information) was increased in p101‐deficient type 2 DC and GM‐CSF‐iDCs (Figure S6B–E, Supporting Information), which may explain the increase in the antigen cross‐presentation of IC (Figure 2E,F), although the up‐take of IgG‐coated beads has not been affected by p101 deletion (Figure 3E). The protein levels of the costimulatory molecules CD40 and CD80 were not reduced in p84‐ or p101‐deficient DCs (Figure S6F,G, Supporting Information), whereas the protein level of CD86 was slightly, but significantly reduced in p101‐deficient type 2 DCs. Finally, as expected from the FACS experiments showing a normal number of cells in the absence of p84 and p101 under basal conditions, the major transcription factors and genes involved in DC differentiation were not affected in the absence of PI3Kγ units (Figure S5B,C and Table S1, Supporting Information). Since the expression of the FcγR and costimulatory molecules was not decreased in p84‐deficient DCs, we hypothesized that in splenic type 2 DCs the p110/p84 complex of PI3Kγ regulates an intracellular step of antigen processing during cross‐presentation of ICs, possibly at a post‐transcriptional step that depends on the p110/p84 complex.

### 2.4 p84 Regulatory Subunit of PI3Kγ Regulates ROS Production by NOX2

A major target of class I PI3K activity is the mTOR complex,^[^ ^26^ ^]^ but mTOR complex activation was not affected by p84 or p101 deletion (Figure S2E,F, Supporting Information). Therefore, we searched for other targets of the class I PI3K enzymes. One such potential target was the nicotinamide adenine dinucleotide phosphate (NADPH) oxidase NOX2, which is regulated by PI3K.^[^ ^36^ ^]^ NOX2 is an enzymatic complex formed by several cytosolic proteins (p47phox, p67phox, and p40phox) and 2 transmembrane proteins (p22phox and gp91phox). The catalytic subunit, gp91phox is recruited to the phagosomal membrane in cDCs and GM‐CSF‐derived iDCs^[^ ^37^, ^38^ ^]^ and generates superoxide anions (O^2−^) in the lumen of phagosomes, which are dismutated in the presence of protons (H^+^) to produce hydrogen peroxide (H_2_O_2_) and other reactive oxygen species (ROS). The consumption of protons during ROS production keeps the phagosomal pH neutral, which reduces proteolytic activity in the phagosomes and thus facilitates cross‐presentation.^[^ ^38^ ^]^ The important role of pH neutralization induced by superoxide anion dismutation in cross‐presentation has also been demonstrated in plasmacytoid DCs (pDCs), which acquire cross‐presentation ability after stimulation by Toll‐like receptor (TLR) ligands. In contrast to cDCs and GM‐CSF‐derived iDCs, pDCs use mitochondrial ROS to support cross‐presentation.^[^ ^39^ ^]^ Although ROS production has been shown to be involved in the cross‐presentation efficacy of soluble antigens and antigen‐coated beads, its role in the cross‐presentation of ICs has not been tested. To evaluate the involvement of ROS in the cross‐presentation of ICs, we first used a broad NOX inhibitor, diphenylene iodonium (DPI), which completely blocked the cross‐presentation of ICs by type 2 DCs (Figure S7A,B, Supporting Information). This result showed that ROS production in GM‐CSF iDCs is required for efficient cross‐presentation of ICs.

**Figure 4:**
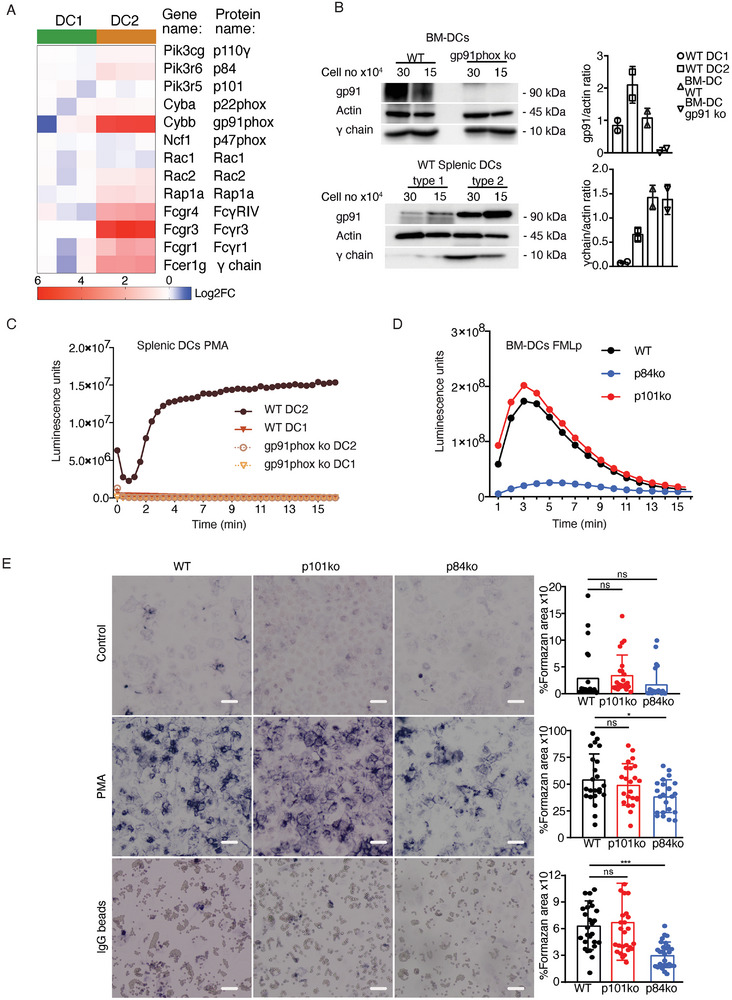
p84 regulatory subunit of PI3Kγ controls ROS production in type 2 DCs and GM‐CSF BM‐derived iDCs. A) Heat map showing the differential expression of the genes encoding the FcγRs, PI3Kγ subunits, and Nox2 NADPH subunits (Rap1, Rac2, Rac1, p47phox, p22phox, gp91phox) in murine splenic conventional DCs, as assessed by bulk RNA sequencing (Log2 FC). B) Expression of gp91phox subunit of NOX2 complex and of FcRs γ‐chain in WT and gp91phox‐deficient conventional and GM‐CSF‐derived iDCs was analyzed by immunoblot. Actin was used as a loading control. The blots are representative of 2 independent experiments. C) Type 1 and 2 splenic conventional DCs were isolated by cell sorting from WT and gp91phox‐deficient mice and their ability to produce ROS after PMA stimulation was measured by luminol‐amplified chemiluminescence. Data are representative of 2 independent experiments. D) WT, p84, and p101‐deficient GM‐CSF‐derived iDCs at day 7 were tested for their ability to produce ROS after the activation of Formyl peptide receptor (FPR) with N‐Formylmethionyl‐leucyl‐phenylalanine (fMLP), as in (C). Data are representative of 3 independent experiments. E) WT, p84, and p101‐deficient iDCs were differentiated in vitro with GM‐CSF from bone marrow precursors. On day 7 they were treated with IgG‐coated beads and the ROS production was assessed by the formation of formazan crystals. As a positive control, PMA‐treated cells were used. Quantification of the signal was performed using ImageJ software. Each dot represents the quantification of a different microscope field. Scale bars = 30 µm. Data are pooled from 2 independent experiments (*: 0.01 < *p* < 0.05; ***0.0001 < *p* < 0.001; one‐way ANOVA test followed by Dunnett's multiple comparisons).

The production of ROS in the phagocytic pathways is due to the activity of gp91phox, according to previous reports^[^ ^38^, ^40^ ^]^ Although these reports showed that gp91phox controls phagosomal ROS in both type 1 and type 2 DCs, based on RNA sequencing data, the expression of the gp91phox complex must be much higher in type 2 DCs and correlated with the expression of FcγR and p84 (**Figure** **4**A). We confirmed these data by immunoblotting and showed that NOX2 components and the γ chain of the FcγR are well expressed in type 2 DCs and GM‐CSF‐derived iDCs, but barely detectable in type 1 DCs (Figure 4B; Figure S7C, Supporting Information).

As expected from the expression pattern of NOX2 complex members, only type 2 DCs produced ROS when cells were stimulated with phorbol 12‐myristate 13‐acetate (PMA), a direct activator of protein kinase C (PKC) (Figure 4C; Figure S7D, Supporting Information). Activation of PKC is directly linked to NOX2 assembly and ROS production via phosphorylation of the NOX2 subunit p47phox. In addition to PKC, other kinases such as protein kinase B (PKB/AKT) and ERK1/2 are capable of phosphorylating p47phox. Phosphorylation of p47phox changes the conformation of the protein and makes it accessible for protein‐lipid interactions via the SH3 and PX domains. p47phox can thus bind to p22phox via its SH3 domain^[^ ^41^ ^]^ and to phosphatidylinositol‐3‐phosphate‐rich membranes via its PX domain.^[^ ^42^ ^]^ These structural features of p47phox, which are essential for the assembly of the NOX2 complex, explain how class I PI3Ks can activate NOX2. Class I PI3Ks increase the phosphoinositide content of cellular membranes and activate kinases capable of phosphorylating p47phox, including PKC.^[^ ^43^ ^]^

To investigate if the regulatory subunits of PI3Kγ are involved in NOX2 assembly and ROS production in GM‐CSF‐derived iDCs, we used fMLP (formyl‐Met‐Leu‐Phe) peptide, a Formyl peptide receptor (FPR) agonist known to activate PI3K.^[^ ^44^ ^]^ Under these conditions, the p84‐deficient GM‐CSF‐derived iDCs were unable to produce ROS (Figure 4D; Figure S7E, Supporting Information). To measure ROS production downstream of FcγR activation, we performed an IgG‐coated bead phagocytosis assay and measured ROS using the NBT colorimetric assay for ROS production. GM‐CSF‐derived iDCs deficient for p84 showed significantly lower levels of ROS production compared to WT and p101‐deficient cells (Figure 4E). This difference was much smaller when we activated the cells with PMA, a direct activator of PKC, but still significant because the presence of PI3Ps in the endosomal membrane is essential for efficient interaction of the p67phox subunit of NOX2 with other members of the NOX2 complex^[^ ^45^ ^]^ (Figure 4E).

These results demonstrate that the p84 subunit of PI3Kγ is required for ROS production. Probably by facilitating NOX2 assembly, because at the transcriptional level, the expression of NOX2 pathway factors was not different between WT cells and those deficient in PI3Kγ regulatory subunits (Figure S5D, Supporting Information).

### 2.5 The Lipid Kinase Activity of PI3Kγ is Required for ROS Production and ICs Cross‐Presentation

**Figure 5:**
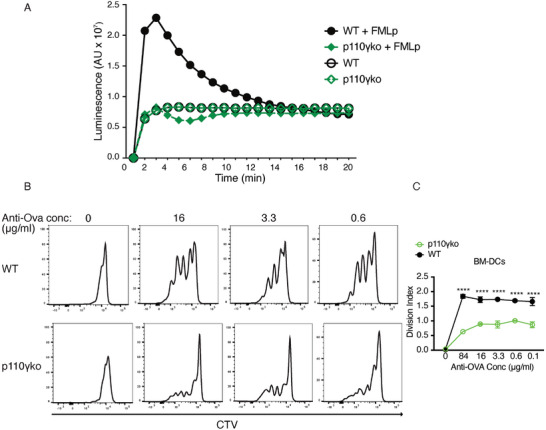
Enzymatic activity of PI3Kγ is required for ROS production and immune complexes cross‐presentation. A) WT and p110γ‐deficient GM‐CSF‐derived iDCs were differentiated in vitro with GM‐CSF for 7 days. Their ability to produce ROS after the activation of the Formyl peptide receptor (FPR) with N‐Formylmethionyl‐leucyl‐phenylalanine (FMLp) was measured by luminol‐amplified chemiluminescence. Data are representative of 2 independent experiments. B) WT and p110γ‐deficient GM‐CSF‐derived iDCs incubated for 16h with Ova anti‐Ova immune complexes were used to stimulate naïve OT1 T cells. The OT1 T cell activation was measured by CTV dye dilution. C) The graph shows the division index of activated T cells corresponding to panel B. Data are representative of 2 experiments realized in duplicate (****p < 0.0001; two‐way ANOVA test followed by Sidak's multiple comparisons).

Our results showed that the p84 regulatory subunit of PI3Kγ is involved in IC cross‐presentation and ROS production in GM‐CSF‐derived iDCs and type 2 DCs, suggesting that the enzymatic complex formed by p84 and p110γ is required for NOX assembly in these cells. To investigate whether the enzymatic kinase activity of the p84/p110γ complex is involved in ROS production, we tested the ability of GM‐CSF‐derived iDCs lacking the kinase subunit p110γ to produce ROS. While WT cells produced significant amounts of ROS upon activation of their FPRs by fMLP, ROS production under these conditions was abolished in p110γ‐deficient GM‐CSF‐derived iDCs (**Figure** **5**A; Figure S7F, Supporting Information). In addition, the cross‐presentation of Ova antigen from IC was also significantly reduced in the absence of PI3Kγ catalytic activity, due to p110γ deletion (Figure 5B,C; Figure S7G, Supporting Information). We concluded that the activity of the p84/p110γ enzyme complex activates NOX2 in type 2 DCs and GM‐CSF‐derived iDCs and that ROS production in these cells enhances their cross‐presentation ability.

## 3 Discussion

In the field of antigen presentation, the generally accepted concept is that type 1 DCs are the best‐qualified myeloid cells for cross‐presentation. At the same time, several reports have shown that other types of DCs are also very good at cross‐presentation, especially for specific forms of antigen. Among these specific antigen forms, one of the most relevant situations is the cross‐presentation of antigens contained in ICs. The cross‐presentation of ICs has an important impact on the efficacy of immunotherapies based on anti‐tumor mAbs.^[^ ^21^ ^]^

In the context of antitumor mAb therapy, it has been shown that in addition to antibody‐dependent cytotoxicity, mAb therapy induces priming of anti‐tumor cytotoxic T cells by a poorly understood subtype of antigen‐presenting cells. This antibody‐activated cytotoxic T‐cell immune response is responsible for long‐term tumor eradication, at least in mouse models.^[^ ^21^ ^]^ Also, tertiary lymphoid structures inside the tumor microenvironment are believed to foster B‐cell‐dependent antitumor immunity, perhaps via the formation of ICs and cross‐priming of tumor‐specific CD8^+^ T cells.^[^ ^46^ ^]^

We tried to find out which type of DCs are involved in the antigen presentation of ICs by using mice deficient in the type 1 DC subset. The study of mice lacking type 1 DCs has shown that type 2 DCs can prime endogenous T cells and drive the T cell response toward a memory phenotype. We then investigated which signaling pathway, downstream of the Fc receptors activated by ICs, enables type 2 DCs to cross‐present ICs. Our results showed that the p84/p110 complex of PI3Kγ is essential for FcγR‐mediated and antibody‐dependent cross‐presentation.

This type of cross‐presentation occurs via FcγR on DCs interacting with soluble and particulate antigens bound to multiple antibodies. Antibody‐FcγR interaction results in higher antigen uptake,^[^ ^16^, ^47^ ^]^ and a more efficient cross‐presentation than that of free, soluble antigens.^[^ ^23^ ^]^ Modulation of FcγR‐mediated antigen cross‐presentation efficiency is particularly relevant for those APC that cross‐present free antigens poorly, such as iDCs and type 2 DCs. The contribution of these cells to CD8^+^ T cell priming does not appear to be important in steady‐state, when conventional type 1 DCs are by far the most qualified cells for cross‐presentation, whereas type 2 DCs excel in induction of CD4 T cell responses.^[^ ^48^, ^49^, ^50^ ^]^ In contrast, under inflammatory conditions, antigen cross‐presentation by iDCs^[^ ^51^ ^]^ and type 2 DCs can contribute to CD8^+^ T cell activation.^[^ ^52^, ^53^ ^]^ In the case of type 2 DCs, inducible cross‐presentation has recently been shown to be dependent on FcγRI expression.^[^ ^54^ ^]^ In addition to inflammatory conditions associated with infection, FcγR‐mediated cross‐presentation is important for CD8^+^ T cell priming following immunotherapy with antitumor mAbs, when innate immune cells first kill tumor cells by ADCC and in a second step, DCs cross‐present tumor antigens in an FcγR‐dependent manner, leading to CD8^+^ T cell priming and long‐term adaptive immune responses against tumor cells.^[^ ^21^, ^55^ ^]^ Although the DC subset that performs FcγR‐mediated cross‐presentation of tumor cells opsonized by mAbs has not been identified, the ability of type 1 DCs to cross‐present ICs in the absence of FcγR^[^ ^25^ ^]^ suggests that type 2 DCs or other APCs play an essential role in this process, which has been shown to require FcγR expression.^[^ ^21^, ^55^ ^]^

Understanding the importance of downstream FcγR signaling for antigen cross‐presentation has been hampered by the difficulty of identifying the correct experimental setting. For example, early studies used mice lacking the ITAM‐bearing Fc receptor‐associated γ chain encoded by the *FcgeR1* gene. Inflammatory DCs or type 2 DCs from these mice were unable to cross‐present ICs.^[^ ^23^, ^25^, ^56^ ^]^ However, in the absence of the γ chain, the expression of FcγRs at the plasma membrane is strongly reduced, resulting in poor uptake of ICs by DCs. The unambiguous demonstration of the essential role of ITAM‐mediated signaling in the cross‐presentation of ICs came later from the analysis of NOTAM mice, in which the γ chain is well expressed, but the ITAM signaling is abolished by targeted mutation of the 2 tyrosine residues of the ITAM.^[^ ^31^ ^]^ These mice have a normal level of FcγR expression at the plasma membrane and their cells are able to internalize ICs, albeit with lower efficacy than WT counterparts. However, the cross‐presentation of ICs by NOTAM GM‐CSF‐derived iDCs and type 2 DCs is completely abolished, even at high antigen concentrations, demonstrating that the γ chain ITAM signaling is critical for cross‐presentation of ICs.

The need for intact Fc receptor signaling has been attributed to the FcγR‐induced, Syk‐dependent maturation of DCs, which leads to the upregulation of costimulatory molecules and production of pro‐inflammatory cytokines.^[^ ^23^, ^31^, ^57^, ^58^, ^59^ ^]^ Our results indicate that an additional mechanism by which FcγR signaling promotes immune complex cross‐presentation is the increase in ROS production in GM‐CSF‐derived iDCs and type 2 splenic DCs. These results are consistent with previous work showing that restoring DC maturation in NOTAM mice could not correct the observed IC cross‐presentation defect.^[^ ^31^ ^]^

Our results are also consistent with the previously published role of NOX2 and ROS in the cross‐presentation ability of GM‐CSF‐derived iDCs^[^ ^37^, ^38^ ^]^ and the demonstration that type 1 DCs cross‐present ICs in an FcγR‐independent manner.^[^ ^25^ ^]^ The novelty of our study consists in the identification of the lipid kinase PI3Kγ as a central player in ROS production by type 2 DCs and GM‐CSF‐derived iDCs, an important finding considering the increasing efforts to develop PI3Kγ inhibitors and the ongoing trials using these inhibitors in cancer, allergic and inflammatory diseases.^[^ ^26^, ^60^ ^]^

Like the PI3Kδ, PI3Kγ is a member of the class I PI3K family whose expression, under physiological conditions, is much higher in the immune system than in other tissues. Unlike PI3Kδ, PI3Kγ is the only member of the class I PI3K family that has 2 regulatory subunits, p84 and p101, with distinct expression patterns. While p101 expression is ubiquitous in the immune system, the expression of p84 expression is restricted to the myeloid lineage, with the highest expression in monocytes, macrophages, and DCs.^[^ ^61^ ^]^ The relative roles of these regulatory subunits have not been previously studied in immune cells, except in neutrophils, where the p101/p110 complex regulates cell migration and p84/p110 complex controls ROS production.^[^ ^34^ ^]^ The neutrophil example suggests that in immune cells where both regulatory subunits are co‐expressed, they may regulate different functions.

In this study, we have shown that both p84 and p101 regulatory subunits are expressed in GM‐CSF‐derived iDCs and cDCs (Figure 2B,C) and that their function is not redundant. Thus, the p84 regulatory subunit is involved in the cross‐presentation of ICs by GM‐CSF‐derived iDCs and type 2 DCs, a process in which the p101 regulatory subunit did not appear to play a role (Figure 2E–H), but the lipid kinase activity of the PI3Kγ complex was essential (Figure 5). The development of splenic DCs and GM‐CSF‐derived iDCs, antigen uptake, MHC‐I expression and the ability of DCs pulsed with SIINFEKL peptide to activate OT1 transgenic T cells were not affected by the deletion of any of the PIK3γ regulatory subunits (Figure 3). In contrast, the ability of type 2 DCs and GM‐CSF‐derived iDCs to produce ROS was significantly reduced in the absence of the p84 regulatory subunit (Figure 4), demonstrating, that these DCs, similar to neutrophils,^[^ ^34^ ^]^ require the p84/p101 complex of PI3Kγ for ROS production. As previously published for GM‐CSF‐derived iDCs,^[^ ^38^ ^]^ ROS production by type 2 DCs also required NOX2 expression (Figure 4C), and NOX2 activity was essential for the cross‐presentation of ICs by these cells (Figure S7A,B, Supporting Information).

In addition to its role in antigen cross‐presentation, the PIK3γ activity has been demonstrated to participate in DC development in the lung.^[^ ^35^ ^]^ Our results demonstrated that in basal conditions neither the p84/p110, nor the p101/p110 PIK3γ complexes are required for splenic DC development (Figure S2, Supporting Information). However, upon Flt3L injection, mice deficient for regulatory subunits displayed a slight but significant decrease in type 1 DC (Figure S3, Supporting Information). This can be relevant in the case of treatment using Flt3L, which, through induction of type 1 DCs renders melanoma cells more sensitive to immune checkpoint inhibitors.^[^ ^62^ ^]^

Although undeniable experimental evidence has demonstrated the superiority of type 1 DCs in cross‐priming, it is becoming increasingly clear that type 2 DCs can also prime CD8 T cells under certain conditions, such as under type I IFN exposure. Thus, in the context of viral infections, type 2 DCs exposed to type I IFN cross‐present IC containing viral antigens and prime CD8 T cells.^[^ ^62^ ^]^ Additional experimental evidence suggests that type 2 DC also contributes to antitumor defense. For example, the analysis of type 2 DC infiltration in human cancer data sets has shown a significant correlation with survival, with the benefit being seen in tumors that are resistant to control by cytotoxic T cells.^[^ ^30^, ^63^ ^]^ Whether type 2 DCs are involved in this survival via tumor antigen presentation has not been established, although it has been shown in murine models that MHC class I‐dressed type 2 DCs can prime CD8 T cells in the presence of type I IFNs.^[^ ^64^ ^]^ Furthermore, it is not known whether DC2‐mediated cross‐presentation of ICs after mAb therapy plays a role in the induction of cytotoxic T responses. Currently, these studies are hampered by the ability to specifically delete type 2 DC in mice in the absence of a DC2‐specific promoter.

The role of PI3Kγ in the cross‐presentation of ICs that we have demonstrated here suggests that the use of PI3Kγ inhibitors should be carefully evaluated in clinical situations where T cell priming by DCs is important, such as in antitumor therapy.^[^ ^21^ ^]^ PI3Kγ inhibition has been proposed for the treatment of leukemia and lymphoma, where PI3Kγ activity promotes leukemic cell growth, and in breast tumors, where PI3Kγ signaling in tumor‐associated macrophages induces a transcriptional immunosuppressive program.^[^ ^65^ ^]^ In both of these conditions, the effect of PI3Kγ impact was studied in mice deficient in the enzymatic subunit p110γ and it is not possible to predict which regulatory subunit of PI3Kγ is involved in tumor growth or macrophage‐induced immunosuppression. Since our study demonstrated that the antibody‐mediated cross‐presentation is entirely dependent on the p84/p110γ complex of PI3Kγ, it would be important to elucidate which of the 2 PI3Kγ complexes drives tumor growth or immunosuppression, to evaluate whether selective inhibition of the p101/p110γ complex is effective in affecting tumor growth and macrophage‐driven immunosuppression without inhibiting antibody‐dependent cross‐presentation.

## 4 Experimental Section

### Mice

C57BL/6 WT mice were purchased from Envigo. XCR1‐DTA mice were generated by crossing homozygote XCR1^[^ ^66^ ^]^ and Rosa26^lox‐stop‐lox‐DTA^.^[^ ^67^ ^]^ Mice deficient for the PI3Kγ subunits p101, p84, and p110γ were previously described.^[^ ^34^, ^68^, ^69^ ^]^ RAG2‐deficient OT1 T cell receptor transgenic mice were a generous gift from Olivier Lantz (Curie Institut, Paris). All mice used were between 8 and 16 weeks old and were matched for age and sex. All mice were housed and maintained in the CRI U1149 conventional animal facility in individually ventilated cages. All animal experiments were licensed by the “Comité d'éthique” Paris‐Nord/N°121 under the project number APAFIS 12517 and additionally approved by the CRI U1149 Ethical Committee.

### Cell Culture

GM‐CSF‐derived iDCs (BM‐DCs) were produced in vitro by culturing bone marrow precursor cells from large bones for 7 days in a complete medium (IMDM complemented with 10% FCS, 2 mM glutamine, 100 U mL^−1^ penicillin, 100 µg mL^−1^ streptomycin, 50 µM b‐mercaptoethanol supplemented with 20 µg mL^−1^ GM‐CSF). BM‐DC differentiation was analyzed by staining with CD11c, MHC‐II, and CD11b antibodies as described previously.^[^ ^70^ ^]^

Melanoma cell line B16 cells secreting Flt3‐L were cultured in complete RPMI medium (RPMI supplemented with 10% FCS, 2 mM glutamine, 100 U mL^−1^ penicillin, 100 µg mL^−1^ streptomycin, 50 µM b‐mercaptoethanol). The melanoma cells were split every 48 h, checked for viability with Trypan Blue, and 5 to 10 million live cells in sterile PBS were injected subcutaneously.

For the co‐culture of primary DCs and T cells used in cross‐presentation assays, complete RPMI was also used.

### Antibodies

The following antibodies were used in this study:

*Immunoblotting*: Commercial antibodies: mouse monoclonal anti beta‐Actin (clone AC‐15; Sigma; dilution 1:10000), mouse monoclonal anti‐p110γ (clone D12, Santa Cruz; dilution 1:1000), rabbit polyclonal anti‐p101 (Cell Signaling Technologies; dilution 1:1000), mouse monoclonal anti‐gp91phox coupled to horse radish peroxidase (HRP) (clone 54.1; Santa Cruz, dilution 1:200), rabbit monoclonal anti‐pAkt (S473) (clone D9E; Cell Signaling Technologies, dilution 1:2000), rabbit polyclonal anti‐Akt (Cell Signaling Technologies; dilution 1:1000), rabbit monoclonal anti‐pmTOR (S2448) (clone P9C2; Cell Signaling Technologies; dilution 1:1000), rabbit monoclonal anti‐mTOR (clone 7C10; Cell Signaling Technologies; dilution 1:1000). Anti‐FcRγ chain rabbit polyclonal antibody was a gift from Ulrich Blank (U1149, INSERM, Paris).^[^ ^71^ ^]^ Rabbit polyclonal antibody against p84 was produced for our laboratory by Covalab (France) by immunization of 2 rabbits with His‐tagged recombinant murine p84 produced in *E Coli*. After the purification via Protein G‐sepharose binding of total IgGs from immunized mice, the anti‐p84 specific IgGs were purified using recombinant p84 coupled to CNBr‐Activated Sepharose 4B (GE Healthcare) and it was used at a final concentration 5 µg mL^−1^. All secondary antibodies were goat anti‐species coupled with HRP (Jackson ImmunoResearch).

*Flow cytometry*: GM‐CSF‐derived iDCs were stained with mouse anti‐mouse H‐2K^b^ (clone AF6‐88.5; Biolegend), rat anti‐mouse I‐A/I‐E‐PE (clone M5/114.152; Biolegend); rat anti‐mouse CD11b‐FITC (clone M1/70; BD Biosciences) or rat anti‐H2‐Kb‐A488 (clone AF6‐88.5; Biolegend). Enriched DCs from splenocytes were stained with rat anti‐mouse I‐A/I‐E‐PE (clone M5/114.152; Biolegend), Armenian hamster anti‐mouse CD11c‐APC/Cy7 or BV650 (clone N418; Biolegend); mouse anti‐mouse XCR1‐PerCP/Cy5.5 or APC (clone ZET; Biolegend) and rat anti‐mouse CD172a (SIRPα)‐APC or FITC (clone P84; Biolegend). OT1 T cells were stained with rat anti‐mouse CD8α‐APC (clone 53–6.7; Biolegend) and rat anti‐mouse TCR Vα2‐PE (clone B20.1; Biolegend). Dead cells were excluded by using either 7AAD viability staining solution (dilution 1:100) (420404, Biolegend) or Ghost Dye Violet 510 (TONBO Biosciences; dilution 1:500).

The antibodies used in the in vivo experiments, co‐stimulatory molecules expression analysis, and FcγRs expression analysis are described below.

*Antigen cross‐presentation assays*: Ovalbumin‐specific rabbit polyclonal serum (Sigma) was purified with Protein G Sepharose beads (GE Healthcare), dialyzed against PBS, sterilized by filtration, and stored at −20 °C with 10% glycerol in its final composition.

### Production of Recombinant His‐Tag‐p84

The cDNA coding for p84 was amplified with the following primers 5’‐ CGCGCGGCAGCCATAGCatggagagctcagatgtggag‐3’ (Forward) and 5’‐ GTCATGCTAGCCATAttattggatgatgccagagaatg‐3’ (Reverse) and introduced by homologous recombination into pET28b (+) plasmid (Novagen, Millipore SAS). Bold sequences were designed to allow homologous recombination (GeneArt Seamless Enzyme Mix, Life Technologies SAS). The resulting plasmid pET‐28b‐p84 was verified by sequencing and transformed into Rosetta 2(DE3) Competent Cells (Novagen, Millipore SAS). Rosetta 2 Competent Cells bacteria were grown at 37 °C in LB‐media containing Kanamycin (50µg/mL) and chloramphenicol (25 µg mL^−1^). The production of p84 was induced during 3 h by 0.5 mM β‐d‐1‐thiogalactopyranoside (IPTG) during the mid‐exponential phase at 37 °C. Bacteria were pelleted and lysed by sonication in denaturing buffer (50 mM Tris‐HCl, 300 mM NaCl, SDS 10%, DTT 10 mM). Recombinant p84 was purified using an Äktat start purification system and HisTrap FF Crude resin (Cytiva life sciences) equilibrated with washing buffer (50 mM Tris‐HCl, 300 mM NaCl, 20 mM imidazole). The recombinant protein was eluted by increasing imidazole concentration to 500 mM and used for the production of anti‐p84 rabbit polyclonal antibodies.

### Immunoblotting

Cells were lysed in lysis buffer (50 mM Tris‐HCl pH 7.5, 150 mM NaCl, 1% CHAPS) supplemented with EDTA‐free complete protease inhibitors (11873580001; Sigma‐Aldrich) and phosphatase inhibitors (phosphatase inhibitors cocktail 2; P5726; Sigma‐Aldrich, phosphatase inhibitors cocktail 3; P0044; Sigma‐Aldrich) when needed and were let on ice for 30 min. The cell lysate was cleared by centrifugation at 15 000 g at 4 °C for 15 min. The cell lysates were separated by SDS‐PAGE using Criterion 4%–15% acrylamide gels (BioRad) in Tris‐Glycine‐SDS buffer. The proteins were transferred on PVDF membranes (BioRad) using a Trans‐Blot Turbo Transfer System from BioRad. Membranes were blocked overnight in 4% nonfat milk and incubated for 1 h with each antibody, washed extensively, and incubated for 5 min with Clarity Western ECL Substrate (BioRad). The chemiluminescence signal was acquired using a ChemiDoc Imaging System and the quantification was realized with the ImageLab software (BioRad).

### Splenic DC Isolation

The proliferation of conventional splenic DCs was induced in vivo by injecting 5 to 10 million live Flt3L‐B16 melanoma cells. Ten days later, mice spleens were digested with 200U of Collagenase D (Roche) and 10 µg mL^−1^ DNAse I (Sigma), and CD11c positive cells were enriched using CD11c‐magnetic beads (Miltenyi). After red cell lysis (ACK buffer, 3 min incubation at RT) the CD11c cells were stained with the following panel of antibodies: rat anti‐mouse I‐A/I‐E‐PE; Armenian hamster anti‐mouse CD11c‐APC/Cy7; mouse anti‐mouse XCR1‐APC and rat anti‐mouse SIRPα‐FITC. The cells were then sorted on the BD FACSAria‐II Cell Sorter (BD Biosciences). Type 1 DCs were CD11c^hi^, MHC‐II^hi^, and XCR1^+^, while type 2 DCs were CD11c^hi^, MHC‐II^hi^ and SIRPα^+^. For the ex vivo cross‐presentation assay, no Flt3L‐B16 cells were injected. Splenic DCs were enriched using the negative selection pan dendritic cell Isolation kit (130‐100‐875, Miltenyi) and stained with anti‐I‐A/I‐E‐PE, anti‐XCR1‐PerCP‐Cy5.5 and anti‐SIRPα‐APC. The type 2 DCs were sorted using the BD Melody Cell Sorter (BD Biosciences).

### BMDC Culture

Murine bone marrow‐derived DCs (BMDCs) were produced in vitro by culturing cells extruded from large bones for 6–8 days in a complete medium (Iscove's modified Dulbecco's medium (IMDM) complemented with 10% fetal calf serum (FCS), 2 mM glutamine, 100 U mL^−1^ penicillin, 100 g mL^−1^ streptomycin, 50 mM β‐mercaptoethanol supplemented with J558 supernatant containing 20 µg mL^−1^ granulocyte‐macrophage colony‐stimulating factor (GM‐CSF)).

### Transgenic OT1 T Cells Isolation

OT1 T cells were isolated with negative selection CD8 T cell kit (Miltenyi) from lymph nodes (inguinal and mesenteric) and the spleen of transgenic RAG2 ko‐OT1 transgenic mice and labeled with 5 mM Cell Trace Violet (CTV) reagent (Thermo Fisher) for 20 min at 37 °C in PBS. After 2 washes with a complete medium, the labeled T cells were used for cross‐presentation assays or adoptive transfer in mice.

### In Vitro Antigen Cross‐Presentation Assay

Ova/anti‐Ova ICs were formed by 1h incubation at 4°C of 20 µg mL^−1^ Ova with serial dilutions (between 420 and 0.004 µg mL^−1^) of anti‐Ova rabbit IgG. ICs were then incubated with 50 000 spleen DCs or 100 000 GM‐CSF‐derived iDCs in 200 µL of complete medium overnight. The next day, the DCs were washed twice and 200 000 transgenic CTV‐labeled OT1 T cells were added to the culture.

### Ex Vivo Antigen Cross‐Presentation Assay

300 µg Ova were incubated with 900 µg anti‐OVA for 30 min at 37 °C. The formed ICs were then injected intraperitoneally in mice. As a control, 300 µg of Ova were injected in p101‐deficient mice. After 3 h the mice were sacrificed and splenic cDC2 was isolated as described above. 25 000 cDC2 were co‐cultured with 50 000 CTV‐labeled OT1 T cells at 37°C for 72 h.

### Measurement of T Cell Activation

T cell proliferation was evaluated after 3 days of incubation with cross‐presenting DCs. Cell trace Violet dye dilution was measured by flow cytometry using the BD LSRFortessa cell analyzer (BD Biosciences). The division index of activated OT1 cells was calculated according to Angulo et al.^[^ ^72^ ^]^ The OT1 T cell activation by SIINFEKL‐pulsed DCs was also evaluated at 72 h by flow cytometry or at 24 h by measuring IL‐2 concentration in supernatants by sandwich ELISA, using Nunc Maxisorp plates and the following antibodies: rat anti‐mouse IL‐2 (clone JES6‐1A12) for IL‐2 capture and rat anti‐mouse IL‐2/Biotin (clone JES6‐5H4; both BD Biosciences) for IL‐2 detection. Signal amplification reagents were streptavidin/HRP (Thermo Scientific) and OptEIA TMB substrate (BD Biosciences).

### In Vivo Antigen Cross‐Presentation Assay

For the in vivo adoptive transfer experiment, 4 × 10^5^ CTV‐labeled OT1 T cells were injected intravenously in WT or XCR1‐DTA mice. The next day Ova‐anti‐Ova ICs (see ex vivo antigen cross‐presentation) were injected intraperitoneally in the mice bearing the OT1 T cells. After 3 days the mice were euthanized, and their spleen was collected. After mechanical disruption of the spleen, rec cell lysis was performed as described above. 10% of the recovered splenocytes were used for T cell proliferation analysis. To gate onto OT1 T cells, the cells with SIINFEKL‐pentamers‐PE (ProImmune) for 30 min at room temperature were stained. Then Fc blockage for 15 min at 4 °C and extracellular staining for 30 min at 4 °C were performed. For the extracellular marker staining and lineage exclusion the following antibodies were used: rat anti‐mouse CD8a‐AF700 (clone KT15; Bio‐rad), rat anti‐mouse CD62L‐BV785 (clone MEL‐14; Biolegend), rat anti‐mouse CD44‐APC/Fire 750 (clone IM7; Biolegend), rat anti‐mouse CD4‐FITC (clone H129.19; Biolegend), rat anti‐mouse CD11b‐FITC (clone M1/70; Biolegend), rat anti‐mouse B220‐FITC (clone RA3.682; eBioscience) and rat anti‐mouse I‐A/I‐E‐FITC (clone M5/114.152; Biolegend). For dead cell exclusion 7AAD was used and to calculate the absolute number of OT1 T cells, counting beads (PCB100; Life Technologies) was used. The flow cytometry analysis was performed in a BD LSRFortessa cell analyzer.

### Bulk RNA‐Sequencing

For the bulk RNA‐sequencing in murine splenic type 1 and type 2 DCs, WT, p84, and p101‐deficient mice were used. As in the ex vivo cross‐presentation assay, no Flt3L‐B16 cells were injected. The mice were euthanized 5 h post injection and their spleens were collected. DCs were enriched in the cell suspension with CD11c positive selection as described above. In each group, 2 mice were used and their DCs were pooled before cell sorting. For each condition, it had 3 replicates. For each replicate (*n* = 3) 10.000 cells were sorted. Type 1 DCs were sorted only from the WT mice. Type 1 and type 2 DCs were directly sorted in 150 µL of TCL lysing buffer (Qiagen) containing 1% β‐mercaptoethanol and stored immediately at −80 °C till RNA extraction. RNA was extracted using the Single Cell RNA purification kit (Norgen, Cat#51800) according to the manufacturer's instructions. After extraction, total RNA was analyzed on the Agilent 2100 BioAnalyzer System. RNA quality was estimated with RNA Integrity Number (RIN). RNA sequencing libraries were prepared using the SMARTer Stranded Total RNA‐Seq Kit v2 – Pico Input Mammalian (Clontech/Takara). The input quantity of total RNA was varying between 1 and 10 ng per sample. This protocol includes a first step of RNA fragmentation, using a proprietary fragmentation mix at 94 °C. The time of incubation was set up for each sample, based on the RNA quality, and according to the manufacturer's recommendations. After fragmentation, indexed cDNA synthesis was performed. Then the ribodepletion step was performed, using probes specific to mammalian rRNA. PCR amplification was finally achieved to amplify the indexed cDNA libraries, with a number of cycles set up according to the input quantity of tRNA. Library quantification and quality assessment were performed by Qubit fluorometric assay (Invitrogen) with dsDNA HS (High Sensitivity) Assay Kit and LabChip GX Touch using a High Sensitivity DNA chip (Perkin Elmer). Libraries were then equimolarly pooled and quantified by qPCR using the KAPA library quantification kit (Roche). Sequencing was performed on the NovaSeq 6000 (Illumina), targeting between 15 and 20 M reads per sample and using paired‐end 2 × 100 bp.

Analysis of the raw data was performed using an R script to remove all the features for which there were less than 300 mapped reads in total. The filtered data table was used for analysis and visualization with the Phantasus online tool (v. 1.23.8)^[^ ^73^ ^]^ and the integrated limma package normalization and group comparison with default parameters.

### Measurement of ROS Production by Luminol‐Amplified Chemiluminescence

DCs (4 000) were suspended in 0.5 mL Hanks balanced salt solution containing 10 µM luminol (Sigma) and 5 units of HRP (Sigma), preheated at 37 °C. The DCs were stimulated either with 10^−5^ M N‐formyl‐methionyl‐leucyl‐phenylalanine (fMLP) (Sigma) or with 600 ng mL^−1^ phorbol 12‐myristate 13‐acetate (PMA), and the chemiluminescence was recorded using the Biolumat LB937 instrument (Berthold).

### Preparation of IgG‐Coated Beads

50 µL of amino‐beads (polybead amino 3.0‐micron microspheres; 9003‐53‐6; Polysciences) were washed 3 times with PBS and then resuspended in 0.5 mL glutaraldehyde 8% (Sigma). The beads were mixed with the glutaraldehyde solution for 4 h at room temperature. After a wash with PBS, beads were resuspended in 250 µL PBS containing 0.5 mg mL^−1^ polyclonal rabbit IgG (Jackson Immunoresearch) and mixed o/n at 4 °C. The next day, beads were centrifuged in a microcentrifuge for 4 min at 14 000 rpm resuspended in 500 µL of 0.5 m glycine, and mixed gently for 30 min at room temperature to block unreacted sites. Finally, beads were washed twice with PBS and resuspended in their initial volume with PBS. Beads were stored at 4 °C for up to 2 weeks.

For the phagocytosis assay, fluorescent beads were used and were coated with 0.1 mg mL^−1^ rabbit polyclonal IgG in carbonate buffer at pH 8 for 2 h at room temperature. The beads were then blocked with 100 mM glycine and finally resuspended in PBS as described above.

### Measurement of ROS Production in BMDCs by the NBT Assay

BMDCs were prepared as described above. 50 × 10^3^ BMDCs were seeded in fibronectin‐treated microscopy slides and incubated for 1 h at 37 °C to allow cells to attach. Slides were placed in a 24‐well plate and 500 µL medium was added. Cells were incubated o/n at 37 °C. The next day the medium was replaced by 300 µL new medium supplemented with low IgG serum to minimize the activation of the FcγRs. 20 µL of 1:10 diluted IgG‐coated beads were added to the medium and cells were centrifuged for 3 min at 2 000 rpm to allow the beads to attach to the cells. Freshly prepared NBT reagent was added to the medium at a final concentration of 0.25 mg mL^−1^. For positive control, cells treated with 0.325 ng µL^−1^ PMA as it activates directly the PKC was used. After incubation at 37 °C for 1 h cells were fixed MeOH for 10 min at room temperature. Cells were visualized in a Leica DMI 6000 microscope and the images were analyzed with ImageJ software.

### Expression of FcγRs

GM‐CSF‐derived iDCs and splenocytes from WT, p84ko, and p101 mice were prepared as described above. To analyze the expression of the α chain of FcγRs we used 1 × 10^5^ GM‐CSF‐derived iDCs and 1/10 of the total splenocyte suspension. For the GM‐CSF‐derived iDCs, the following antibody panel was used. FcγRs: mouse anti‐mouse CD64‐BV421 (clone X54‐5/7.1; Biolegend), mouse anti‐mouse CD32B‐APC (clone AT130‐2; Biolegend), rat anti‐mouse CD16‐FITC (clone S17014E; Biolegend) and Armenian Hamster anti‐mouse CD16.2‐AF700 (clone 9E9; Biolegend). The corresponding isotype controls with the same conjugates were also purchased from BioLegend. Extracellular markers: rat anti‐mouse I‐A/I‐E‐BV510 (clone M5/114.152; Biolegend), rat anti‐mouse CD11c‐BV650 (clone N418; Biolegend) and rat anti‐mouse CD11b‐BV785 (clone M1/70; Biolegend). For the splenic DCs, the following antibody panel was used. FcγRs: mouse anti‐mouse CD64‐FITC (clone X54‐5/7.1; Biolegend), mouse anti‐mouse CD32B‐APC (clone AT130‐2; Biolegend), rat anti‐mouse CD16‐PE/Dazzle 594 (clone S17014E; Biolegend) and Armenian Hamster anti‐mouse CD16.2‐PE (clone 9E9; Biolegend). The corresponding isotype controls with the same conjugates were also purchased from BioLegend. Extracellular markers: rat anti‐mouse I‐A/I‐E‐BV421 (clone M5/114.152; Biolegend), rat anti‐mouse CD11c‐APC/Cy7 (clone N418; Biolegend), rat anti‐mouse CD11b‐BV785 (clone M1/70; Biolegend), mouse anti‐mouse XCR1‐PerCP/Cy5.5 (clone ZET; Biolegend) and rat anti‐mouse MerTK‐BV711 (clone 2B10C42; Biolegend). First, the cells were stained with the antibodies against the FcγRs for 30 min at 4 °C. In order to eliminate the signal derived from the nonspecific binding of the antibodies via their Fc region, we used as controls 4 different mixes of the antibodies against FcγRs where 1 antibody was replaced with the equivalent isotype control each time. After the incubation, the antibodies against extracellular markers (in 2X concentration) were added to the cells without intermediate washing. The staining was performed at 4 °C for 30 min. After the end of the staining cells were washed twice with FACS buffer and analyzed using the BD LSRFortessa cell analyzer. To exclude the dead cells, 7AAD was used as described above, and counting beads were added too.

### Expression of co‐stimulatory Molecules: GM‐CSF‐Derived iDCs

GM‐CSF‐derived iDCs from WT, p84ko, and p101ko, were differentiated from bone marrow precursors as described above. To analyze the expression of the co‐stimulatory molecules CD40, CD80 and CD86, 1 × 10^5^ GM‐CSF‐derived iDCs were plated in round bottom 96‐well plates in the presence of Ova‐anti‐Ova ICs for 24h. The ICs were prepared as described in the in vitro antigen cross‐presentation assay, with the concentration of Ova at 20 µg mL^−1^ and of anti‐Ova at 84 µg mL^−1^ which was the concentration with the higher effect in the in vitro antigen cross‐presentation assay. Cells were also left untreated to evaluate the expression of the co‐stimulatory molecules at a steady state. The medium contained 20 ng mL^−1^ GM‐CSF. The panel used to stain extracellular markers and co‐stimulatory molecules were the following: rat anti‐mouse CD40‐PE (clone 3/23; Biolegend), Armenian hamster anti‐mouse CD80‐AF488 (clone 16‐10A1; Biolegend), rat anti‐mouse CD86‐PE/Cy7 (clone GL‐1; Biolegend), rat anti‐mouse I‐A/I‐E‐BV510 (clone M5/114.152; Biolegend), rat anti‐mouse CD11c‐BV650 (clone N418; Biolegend) and rat anti‐mouse CD11b‐BV785 (clone M1/70; Biolegend). Before the extracellular staining, the cells were treated with an Fc blocking reagent (dilution 1:200) for 15 min at 4 °C. To exclude the dead cells, 7AAD was used as described above. The cells were analyzed in a BD LSRFortessa cell analyzer.

### Expression of co‐stimulatory Molecules: Splenic Type 2 DCs

To evaluate the expression of co‐stimulatory molecules in a more physiologically relevant way in splenic type 2 DCs, intraperitoneally wt, p84ko, and p101ko mice with Ova‐anti‐Ova ICs as described in the ex vivo antigen cross‐presentation assay was injected. The 1/10 of the splenocytes was used for subsequent analysis and after Fc blockade the cells were stained with the following panel: rat anti‐mouse CD40‐PE (clone 3/23, Biolegend), Armenian hamster anti‐mouse CD80‐AF488 (clone 16‐10A1, Biolegend), rat anti‐mouse CD86‐PE/Cy7 (clone GL‐1), rat anti‐mouse I‐A/I‐E‐BV510 (clone M5/114.152; Biolegend), rat anti‐mouse CD11c‐BV650 (clone N418, Biolegend), mouse anti‐mouse XCR1‐PerCP/Cy5.5 (clone ZET, Biolegend), rat‐anti mouse SIRPα‐APC (clone P84, Biolegend), rat anti‐mouse CD45‐BUV395 (clone 30‐F11, BD Horizon), Armenian hamster anti‐mouse CD3ε‐biotin (clone 145‐2C11, Biolegend), rat anti‐mouse CD19‐biotin (clone 6D5, Biolegend), rat anti‐mouse Gr‐1‐biotin (clone RB6‐8C5, Biolegend), rat anti‐mouse F4/80‐biotin (clone BM8, Biolegend), mouse anti‐mouse NK1.1‐biotin (clone PK130, Biolegend). After washing twice with FACS buffer, the cells were stained with streptavidin‐APC/Cy7 (dilution 1:400) for 20 min at 4 °C. After extensive washing, dead cells were marked with 7AAD. The cells were analyzed using a BD LSRFortessa X‐20 cell analyzer.

All flow cytometry and cell sorting data were analyzed and processed using the Flowjo software.

### Statistical Analysis

Pre‐processing of data was described individually for each type of experiment in the Materials and Methods section. Error bars represent the mean ± SD and the sample size and statistical tests, including their variant (one‐tailed, two‐tailed) are indicated in the Figure legends. Statistical analysis was performed with GraphPad Prism (v. 7) software.

### GEO Submission

The data discussed in this publication have been deposited in NCBI's Gene Expression Omnibus (Edgar et al., 2002) and are accessible through GEO Series accession number GSE273544 (https://www.ncbi.nlm.nih.gov/geo/query/acc.cgi?acc=GSE273544).

## Conflict of Interest

The authors declare that they have no conflict of interest.

## Author Contributions

D.K., A.C.A. P.G., and L.S. contributed equally to this work. L.S., A.C.A., D.K., and P.G. conceived and designed the experiments. ACA performed most of the in vitro cross‐presentation, antigen uptake, and ROS production experiments, D.K. performed the ex‐vivo/in vivo cross‐presentation experiments, Flow cytometry experiments, RNAseq experiments, and ROS production assays, J.E.B. helped with ROS measurements experiments, M.N. helped with FACS and RNASeq experimental set‐up and data analysis, E.B. produced the recombinant p84 protein, R.M. contributed to the strategy of anti‐p84 antibodies production, C.S. participated to RNASeq data analysis. M.L. and M.W.P. generated the p110γ deficient mice. P.G. and P.B. conceived the strategy of conventional DCs sorting strategy and contributed to the design of several in vivo and ex vivo experiments. M.D. provided the XCR1^Cre^ and Rosa26^lox‐stop‐lox‐DTA^ mice. D.K. and L.S. wrote the manuscript, with critical input from P.G. and M.W.P. and editing by M.D. and C.S.

## Supporting information



Supporting Information

Supporting Table S1

## Data Availability

The data that support the findings of this study are available from the corresponding author upon reasonable request.

## References

[1] O. P. Joffre , E. Segura , A. Savina , S. Amigorena , Nat. Rev. Immunol. 2012, 12, 557. 22790179 10.1038/nri3254

[2] E. Gutiérrez‐Martínez , Front. Immunol. 2015, 6, 363. 26236315 10.3389/fimmu.2015.00363 PMC4505393

[3] M. Guilliams , F. Ginhoux , C. Jakubzick , S H. Naik , N. Onai , B U. Schraml , E. Segura , R. Tussiwand , S. Yona , Nat. Rev. Immunol. 2014, 14, 571. 25033907 10.1038/nri3712 PMC4638219

[4] A. Schlitzer , N. McGovern , F. Ginhoux , Semin. Cell Dev. Biol. 2015, 41, 9. 25957517 10.1016/j.semcdb.2015.03.011

[5] A. O. Kamphorst , P. Guermonprez , D. Dudziak , M. C. Nussenzweig , J. Immunol. 2010, 185, 3426. 20729332 10.4049/jimmunol.1001205 PMC3013633

[6] S. Menezes , D. Melandri , G. Anselmi , T. Perchet , J. Loschko , J. Dubrot , R. Patel , E. L. Gautier , S. Hugues , M. P Longhi , J. Y. Henry , S. A. Quezada , G. Lauvau , A.‐M. Lennon‐Duménil , E. Gutiérrez‐Martínez , A. Bessis , E. Gomez‐Perdiguero , C. E. Jacome‐Galarza , H. Garner , F. Geissmann , R. Golub , M. C. Nussenzweig , P. Guermonprez , Immunity 2016, 45, 1205. 28002729 10.1016/j.immuni.2016.12.001 PMC5196026

[7] Z. Liu , H. Wang , Z. Li , R J. Dress , Y. Zhu , S. Zhang , D. De Feo , W. T. Kong , P. Cai , A. Shin , C. Piot , J. Yu , Y. Gu , M. Zhang , C. Gao , L. Chen , H. Wang , M. Vétillard , P. Guermonprez , I. Kwok , L. G. Ng , S. Chakarov , A. Schlitzer , B. Becher , C.‐A. Dutertre , B. Su , F. Ginhoux , Immunity 2023, 56, 1761. 37506694 10.1016/j.immuni.2023.07.001

[8] J. M. den Haan , S. M. Lehar , M. J. Bevan , J. Exp. Med. 2000, 192, 1685. 11120766 10.1084/jem.192.12.1685 PMC2213493

[9] R. S. Allan , C. M. Smith , G. T. Belz , A. L. van Lint , L. M. Wakim , W. R. Heath , F. R. Carbone , Science 2003, 301, 1925. 14512632 10.1126/science.1087576

[10] D. Sancho , O. P. Joffre , A. M. Keller , N. C. Rogers , D. Martínez , P. Hernanz‐Falcón , I. Rosewell , C. R. E. Sousa , Nature 2009, 458, 899. 19219027 10.1038/nature07750 PMC2671489

[11] L. Bougnères , J. Helft , S. Tiwari , P. Vargas , B. H‐J. Chang , L. Chan , L. Campisi , G. Lauvau , S. Hugues , P. Kumar , A. O. Kamphorst , A‐M. L. Dumenil , M. Nussenzweig , J D. MacMicking , S. Amigorena , P. Guermonprez , Immunity 2009, 31, 232. 19699172 10.1016/j.immuni.2009.06.022 PMC2803012

[12] N. M. Kretzer , D. J. Theisen , R. Tussiwand , C. G. Briseño , G. E. Grajales‐Reyes , X. Wu , V. Durai , J. Albring , P. Bagadia , T. L. Murphy , K. M. Murphy , J. Exp. Med. 2016, 213, 2871. 27899443 10.1084/jem.20160597 PMC5154939

[13] D. J. Theisen , J. T. Davidson , C. G. Briseño , M. Gargaro , E. J. Lauron , Q. Wang , P. Desai , V. Durai , P. Bagadia , J. R. Brickner , W. L. Beatty , H. W. Virgin , W. E. Gillanders , N. Mosammaparast , M. S. Diamond , L. D Sibley , W. Yokoyama , R. D. Schreiber , T. L. Murphy , K. M. Murphy , Science 2018, 362, 694. 30409884 10.1126/science.aat5030 PMC6655551

[14] O. Schulz , S. S. Diebold , M. Chen , T. I. Näslund , M. A. Nolte , L. Alexopoulou , Y.‐T. Azuma , R. A. Flavell , P. Liljeström , C. Reis e Sousa , Nature 2005, 433, 887. 15711573 10.1038/nature03326

[15] C. G. Briseño , M. Haldar , N. M. Kretzer , X. Wu , D. J. Theisen , W. KC , V. Durai , G. E. Grajales‐Reyes , A. Iwata , P. Bagadia , T. L. Murphy , K. M. Murphy , Cell Rep. 2016, 15, 2462. 27264183 10.1016/j.celrep.2016.05.025 PMC4941620

[16] M. Guilliams , P. Bruhns , Y. Saeys , H. Hammad , B. N. Lambrecht , Nat. Rev. Immunol. 2014, 14, 94. 24445665 10.1038/nri3582

[17] C. H. K. Lehmann , A. Baranska , G. F. Heidkamp , L. Heger , K. Neubert , J. J. Lühr , A. Hoffmann , K. C. Reimer , C. Brückner , S. Beck , M. Seeling , M. Kießling , D. Soulat , A. B. Krug , J. V. Ravetch , J. H. W. Leusen , F. Nimmerjahn , D. Dudziak , J. Exp. Med. 2017, 214, 1509. 28389502 10.1084/jem.20160951 PMC5413326

[18] J. S. de Bono , S. Y. Rha , J. Stephenson , B. C. Schultes , P. Monroe , G. S. Eckhardt , L. A. Hammond , T. L. Whiteside , C. F. Nicodemus , J. M. Cermak , E. K. Rowinsky , A. W. Tolcher , Ann. Oncol. 2004, 15, 1825. 15550589 10.1093/annonc/mdh472

[19] S. P. Hilchey , O. Hyrien , T. R. Mosmann , A. M. Livingstone , J. W. Friedberg , F. Young , R. I. Fisher , R. J. Kelleher , R. B. Bankert , S. H. Bernstein , Blood 2009, 113, 3809. 19196657 10.1182/blood-2008-10-185280 PMC2670795

[20] S. Park , Z. Jiang , E. D. Mortenson , L. Deng , O. Radkevich‐Brown , X. Yang , H. Sattar , Y. Wang , N. K. Brown , M. Greene , Y. Liu , J. Tang , S. Wang , Y.‐X. Fu , Cancer Cell 2010, 18, 160. 20708157 10.1016/j.ccr.2010.06.014 PMC2923645

[21] D. J. DiLillo , J. V. Ravetch , Cell 2015, 161, 1035. 25976835 10.1016/j.cell.2015.04.016 PMC4441863

[22] T. Yi , J. Li , H. Chen , J. Wu , J. An , Y. Xu , Y. Hu , C. A. Lowell , J. G. Cyster , Immunity 2015, 43, 764. 26453377 10.1016/j.immuni.2015.08.021 PMC4618158

[23] A. Regnault , D. Lankar , V. Lacabanne , A. Rodriguez , C. Théry , M. Rescigno , T. Saito , S. Verbeek , C. Bonnerot , P. Ricciardi‐Castagnoli , S. Amigorena , J. Exp. Med 1999, 189, 371. 9892619 10.1084/jem.189.2.371 PMC2192989

[24] F. Nimmerjahn , J. V. Ravetch , Nat. Rev. Immunol. 2008, 8, 34. 18064051 10.1038/nri2206

[25] J. M. M. den Haan , M. J. Bevan , J. Exp. Med. 2002, 196, 817. 12235214 10.1084/jem.20020295 PMC2194052

[26] B. Vanhaesebroeck , L. Stephens , P. Hawkins , Nat. Rev. Mol. Cell Biol. 2012, 13, 195. 22358332 10.1038/nrm3290

[27] M. D. Blunt , A. J. Steele , Leuk. Res. Rep. 2015, 4, 60. 26500849 10.1016/j.lrr.2015.09.001 PMC4588368

[28] C. Casals , M. Barrachina , M. Serra , J. Lloberas , A. Celada , J. Immunol. 2007, 178, 6307. 17475859 10.4049/jimmunol.178.10.6307

[29] S. Eickhoff , A. Brewitz , M. Y. Gerner , F. Klauschen , K. Komander , H. Hemmi , N. Garbi , T. Kaisho , R. N. Germain , W. Kastenmüller , Cell 2015, 162, 1322. 26296422 10.1016/j.cell.2015.08.004 PMC4567961

[30] R. Noubade , S. Majri‐Morrison , K. V. Tarbell , Front. Immunol. 2019, 10, 1014. 31143179 10.3389/fimmu.2019.01014 PMC6521804

[31] P. Boross , N. van Montfoort , D. A. C. Stapels , C. E. van der Poel , C. Bertens , J. Meeldijk , J. H. M Jansen , J. S. Verbeek , F. Ossendorp , R. Wubbolts , J. H. W. Leusen , J. Immunol. 2014, 193, 5506. 25355925 10.4049/jimmunol.1302012

[32] T. Joshi , J. P. Butchar , S. Tridandapani , Int. J. Hematol. 2006, 84, 210. 10.1532/IJH97.06140 29349684

[33] S. P. Nobs , C. Schneider , A. K. Heer , J. Huotari , A. Helenius , M. Kopf , PLoS Pathog. 2016, 12, e1005508. 27030971 10.1371/journal.ppat.1005508 PMC4816423

[34] A. Deladeriere , L. Gambardella , D. Pan , K. E. Anderson , P T. Hawkins , L. R. Stephens , Sci. Signal 2015, 8, ra8. 25605974 10.1126/scisignal.2005564

[35] S. P. Nobs , C. Schneider , M. G. Dietrich , T. Brocker , A. Rolink , E. Hirsch , M. Kopf , Immunity 2015, 43, 674. 26453378 10.1016/j.immuni.2015.09.006

[36] P. T. Hawkins , K. Davidson , L. R. Stephens , Biochem. Soc. Symp. 2007, 74, 59. 10.1042/BSS0740059 17233580

[37] C. Jancic , A. Savina , C. Wasmeier , T. Tolmachova , J. El‐Benna , P. M.‐C. Dang , S. Pascolo , M.‐A. Gougerot‐Pocidalo , G. Raposo , M. C. Seabra , S. Amigorena , Nat. Cell Biol. 2007, 9, 367. 17351642 10.1038/ncb1552

[38] A. Savina , C. Jancic , S. Hugues , P. Guermonprez , P. Vargas , I. C. Moura , A.‐M. Lennon‐Duménil , M. C. Seabra , G. Raposo , S. Amigorena , Cell 2006, 126, 205. 16839887 10.1016/j.cell.2006.05.035

[39] M. Oberkampf , C. Guillerey , J. Mouriès , P. Rosenbaum , C. Fayolle , A. Bobard , A. Savina , E. Ogier‐Denis , J. Enninga , S. Amigorena , C. Leclerc , G. Dadaglio , Nat. Commun. 2018, 9, 2241. 29884826 10.1038/s41467-018-04686-8 PMC5993805

[40] A. Savina , A. Peres , I. Cebrian , N. Carmo , C. Moita , N. Hacohen , L F. Moita , S. Amigorena , Immunity 2009, 30, 544. 19328020 10.1016/j.immuni.2009.01.013

[41] J. El‐Benna , P. M.‐C. Dang , M. A. Gougerot‐Pocidalo , J. C. Marie , Exp. Mol. Med. 2009, 41, 217. 19372727 10.3858/emm.2009.41.4.058 PMC2679237

[42] T. Ago , F. Kuribayashi , H. Hiroaki , R. Takeya , T. Ito , D. Kohda , H. Sumimoto , Proc. Natl. Acad. Sci. U.S.A. 2003, 100, 4474. 12672956 10.1073/pnas.0735712100 PMC153580

[43] R. S. Frey , X. Gao , K. Javaid , S. S. Siddiqui , A. Rahman , A. B. Malik , J. Biol. Chem. 2006, 281, 16128. 16527821 10.1074/jbc.M508810200

[44] R. Selvatici , S. Falzarano , A. Mollica , S. Spisani , Eur. J. Pharmacol. 2006, 534, 1. 16516193 10.1016/j.ejphar.2006.01.034

[45] C. D. Ellson , S. Gobert‐Gosse , K. E. Anderson , K. Davidson , H. Erdjument‐Bromage , P. Tempst , J. W. Thuring , M. A. Cooper , Z.‐Y. Lim , A. B. Holmes , P. R. J. Gaffney , J. Coadwell , E. R. Chilvers , P. T. Hawkins , L. R. Stephens , Nat. Cell Biol. 2001, 3, 679. 11433301 10.1038/35083076

[46] W. H. Fridman , M. Meylan , G. Pupier , A. Calvez , I. Hernandez , C. Sautès‐Fridman , Immunity 2023, 56, 2254. 37699391 10.1016/j.immuni.2023.08.009

[47] C. Macri , H. Morgan , J. A. Villadangos , J. D. Mintern , Mol. Immunol. 2021, 139, 193. 34560415 10.1016/j.molimm.2021.07.024

[48] D. Dudziak , A. O. Kamphorst , G. F. Heidkamp , V. R. Buchholz , C. Trumpfheller , S. Yamazaki , C. Cheong , K. Liu , H.‐W. Lee , C. G. Park , R. M. Steinman , M. C. Nussenzweig , Science 2007, 315, 107. 17204652 10.1126/science.1136080

[49] S. Burgdorf , A. Kautz , V. Bohnert , P. A. Knolle , C. Kurts , Science 2007, 316, 612. 17463291 10.1126/science.1137971

[50] K. Hildner , B. T. Edelson , W. E. Purtha , M. Diamond , H. Matsushita , M. Kohyama , B. Calderon , B. U. Schraml , E. R. Unanue , M. S. Diamond , R. D. Schreiber , T. L. Murphy , K. M. Murphy , Science 2008, 322, 1097. 19008445 10.1126/science.1164206 PMC2756611

[51] C. Cheong , I. Matos , J.‐H. Choi , D. B. Dandamudi , E. Shrestha , M. P Longhi , K. L. Jeffrey , R. M. Anthony , C. Kluger , G. Nchinda , H. Koh , A. Rodriguez , J. Idoyaga , M. Pack , K. Velinzon , C. G. Park , R. M. Steinman , Cell 2010, 143, 416. 21029863 10.1016/j.cell.2010.09.039 PMC3150728

[52] A. Ballesteros‐Tato , B. León , F. E. Lund , T. D. Randall , Nat. Immunol. 2010, 11, 216. 20098442 10.1038/ni.1838 PMC2822886

[53] E. Ainsua‐Enrich , I. Hatipoglu , S. Kadel , S. Turner , J. Paul , S. Singh , H. Bagavant , S. Kovats , Mucosal. Immunol. 2019, 12, 1025. 31089186 10.1038/s41385-019-0173-1 PMC6527354

[54] C. Bosteels , K. Neyt , M. Vanheerswynghels , M. J. van Helden , D. Sichien , N. Debeuf , S. De Prijck , V. Bosteels , N. Vandamme , L. Martens , Y. Saeys , E. Louagie , M. Lesage , D. L. Williams , S.‐C. Tang , J. U. Mayer , F. Ronchese , C. L. Scott , H. Hammad , M. Guilliams , B. N. Lambrecht , Immunity 2020, 52, 1039. 32392463 10.1016/j.immuni.2020.04.005 PMC7207120

[55] K. Rafiq , A. Bergtold , R. Clynes , J. Clin. Invest. 2002, 110, 71. 12093890 10.1172/JCI15640 PMC151032

[56] D. H. Schuurhuis , N. van Montfoort , A. Ioan‐Facsinay , R. Jiawan , M. Camps , J. Nouta , C. J. M. Melief , J. S. Verbeek , F. Ossendorp , J. Immunol. 2006, 176, 4573. 16585547 10.4049/jimmunol.176.8.4573

[57] K. Akiyama , S. Ebihara , A. Yada , K. Matsumura , S. Aiba , T. Nukiwa , T. Takai , J. Immunol. 2003, 170, 1641. 12574326 10.4049/jimmunol.170.4.1641

[58] T. W. H. Flinsenberg , E. B. Compeer , D. Koning , M. Klein , F. J. Amelung , D. van Baarle , J. J. Boelens , M. Boes , Blood 2012, 120, 5163. 23093620 10.1182/blood-2012-06-434498

[59] N. van Montfoort , S. M. Mangsbo , M. G. M. Camps , W. W. C. van Maren , I. E. C. Verhaart , A. Waisman , J. W. Drijfhout , C. J. M. Melief , J. S. Verbeek , F. Ossendorp , Eur. J. Immunol. 2012, 42, 598. 22488363 10.1002/eji.201141613

[60] J. Zhu , K. Li , Li Yu , Y. Chen , Y. Cai , J. Jin , T. Hou , Med. Res. Rev. 2021, 41, 1599. 33300614 10.1002/med.21770

[61] S. V. Aguilar , O. Aguilar , R. Allan , Nat. Immunol. 2020, 21, 700. 32577013 10.1038/s41590-020-0687-4

[62] H. Salmon , J. Idoyaga , A. Rahman , M. Leboeuf , R. Remark , S. Jordan , M. Casanova‐Acebes , M. Khudoynazarova , J. Agudo , N. Tung , S. Chakarov , C. Rivera , B. Hogstad , M. Bosenberg , D. Hashimoto , S. Gnjatic , N. Bhardwaj , A. K. Palucka , B. D. Brown , J. Brody , F. Ginhoux , M. Merad , Immunity 2016, 44, 924. 27096321 10.1016/j.immuni.2016.03.012 PMC4980762

[63] S. Iwanowycz , S. Ngoi , Y. Li , M. Hill , C. Koivisto , M. Parrish , B. Guo , Z. Li , B. Liu , JCI Insight 2021, 6, e145885. 34283809 10.1172/jci.insight.145885 PMC8492342

[64] E. Duong , T. B. Fessenden , E. Lutz , T. Dinter , L. Yim , S. Blatt , A. Bhutkar , K. D. Wittrup , S. Spranger , Immunity 2022, 55, 308. 34800368 10.1016/j.immuni.2021.10.020 PMC10827482

[65] M. M. Kaneda , K. S. Messer , N. Ralainirina , H. Li , C J. Leem , S. Gorjestani , G. Woo , A. V. Nguyen , C. C. Figueiredo , P. Foubert , M. C. Schmid , M. Pink , D. G. Winkler , M. Rausch , V. J. Palombella , J. Kutok , K. McGovern , K. A. Frazer , X. Wu , M. Karin , R. Sasik , E. E. W. Cohen , J. A. Varner , Nature 2016, 539, 437. 27642729 10.1038/nature19834 PMC5479689

[66] R. Mattiuz , C. Wohn , S. Ghilas , M. Ambrosini , Y. O. Alexandre , C. Sanchez , A. Fries , T.‐P. Vu Manh , B. Malissen , M. Dalod , K. Crozat , Front. Immunol. 2018, 9, 2805. 30564233 10.3389/fimmu.2018.02805 PMC6288293

[67] D. Brockschnieder , Y. Pechmann , E. Sonnenberg‐Riethmacher , D. Riethmacher , Genesis 2006, 44, 322. 16791847 10.1002/dvg.20218

[68] S. Suire , J. Coadwell , G. J Ferguson , K. Davidson , P. Hawkins , L. Stephens , Curr. Biol. 2005, 15, 566. 15797027 10.1016/j.cub.2005.02.020

[69] E. Hirsch , V. L. Katanaev , C. Garlanda , O. Azzolino , L. Pirola , L. Silengo , S. Sozzani , A. Mantovani , F. Altruda , M. P. Wymann , Science 2000, 287, 1049. 10669418 10.1126/science.287.5455.1049

[70] L. Saveanu , O. Carroll , M. Weimershaus , P. Guermonprez , E. Firat , V. Lindo , F. Greer , J. Davoust , R. Kratzer , S. R. Keller , G. Niedermann , P. van Endert , Science 2009, 325, 213. 19498108 10.1126/science.1172845

[71] P. Launay , C. Patry , A. Lehuen , B. Pasquier , U. Blank , R C. Monteiro , J. Biol. Chem. 1999, 274, 7216. 10066783 10.1074/jbc.274.11.7216

[72] R. Angulo , D. A. Fulcher , Cytometry 1998, 34, 143. 9696158

[73] M. Kleverov , D. Zenkova , V. Kamenev , M. Sablina , M. N. Artyomov , A. A. Sergushichev , Elife 2024, 13, e85722. 38742735 10.7554/eLife.85722 PMC11147506

