# Supplementary material for: Specific Requirement of the p84/p110γ Complex of PI3Kγ for Antibody‐Activated, Inducible Cross‐Presentation in Murine Type 2 DCs
Source: Adv Sci (Weinh). 2024 Oct 9;11(44):2401179. doi: 10.1002/advs.202401179 (PMC11600261; doi:10.1002/advs.202401179)
Supplement: Supplementary file 1 — Supporting Information [file ADVS-11-2401179-s001.docx]

**Supplementary Figures:**


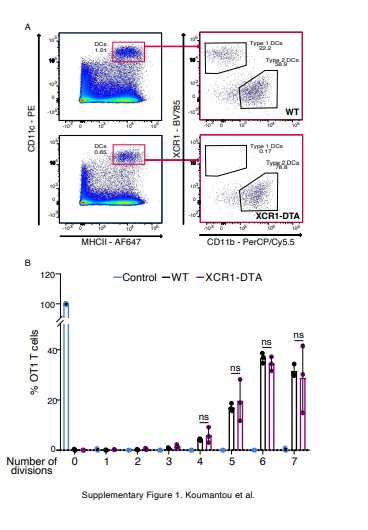


**Supplementary Figure 1: Conventional DC populations and IC cross-presentation in WT and XCR1-DTA mice.**

**(A)** Dot plots showing the staining of splenocytes from naïve WT and XCR1-DTA mice. DCs were gated as MHCII high and CD11c high. Type 1 DCs are considered XCR1^+^CD11b^-^ and type 2 DCs as XCR1^-^CD11b^+^. XCR1-DTA mice were depleted of type 1 DCs as expected.

**(B)** Percentage of OT1 T cells in each division as defined from the MFI of CTV. Each dot represents an individual mouse (ns: non-significant).


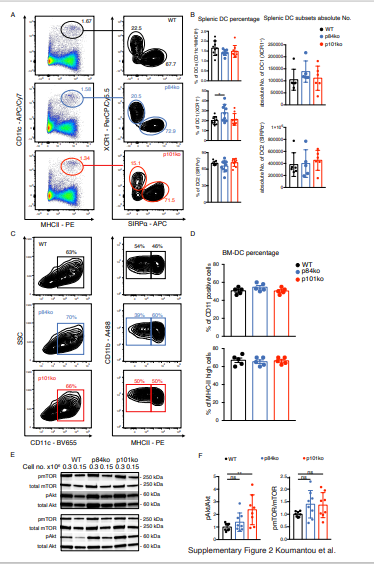


**Supplementary Figure 2. In steady state, the regulatory subunits of PI3K**γ **are not involved in the differentiation of splenic and GM-CSF-derived iDCs.**

**(A)** Flow cytometry analysis of splenic conventional DC in WT, p84 and p101-deficient mice without Flt3L injection. Cells expressing CD11c^hi^ and MHCII^hi^ were defined as dendritic cells, XCR1^+^SIRPα^-^ as type 1 conventional DCs and XCR1^-^SIRPα^+^ as type 2 conventional DCs. The number on the plots are the percentages of each cell population in its parental gate.

**(B)** Graphs (left) showing the percentage of each cell population as described in (A). The experiment was performed in triplicate. Each dot represents an individual mouse. Graphs (right) showing the absolute number of type 1 and type 2 splenic conventional dendritic cells. Results are derived from two individual experiments. Each dot represents an individual mouse (*: 0.01<p<0.05, one-way ANOVA test followed by Dunnett’s multiple comparison method).

**(C-D)** WT, p84 and p101-deficient GM-CSF-derived iDCs were differentiated from bone marrow precursors for 7 days *in vitro* culture with GM-CSF. The cells were stained with anti-CD11c, anti-CD11b and anti-MHC-II antibodies to evaluate their differentiation. The FACS plot is representative of 5 different cultures.

**(E-F)** The steady-state phosphorylation of mTOR and Akt in WT, p84 and p101-deficient GM-CSF-derived iDCs at day 7 of differentiation was analyzed by immunoblot. The data are representative of 4 independent cultures. The graphs in (F) show the signal intensity of phospho-Akt and phospho-mTOR against the total protein. The 8 dots represent the quantification of the two cell amounts deposited on each of the 4 immunoblots. Actin was used as a loading control and all the bands are corrected against it before further analysis (ns: non-significant; **0.001<p<0.01, one-way ANOVA test followed by Dunnett’s multiple comparisons).


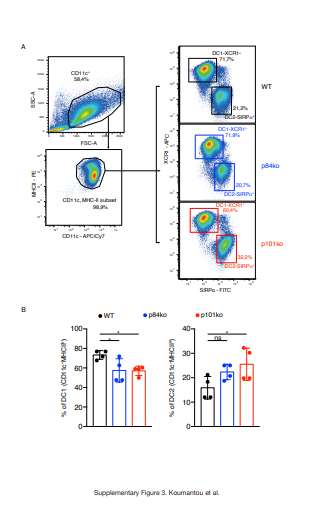


**Supplementary Figure 3. Gating strategy for splenic conventional DC flow cytometry sorting.**

**(A)** The FACS plots represent the gating strategy used for isolation of type 1 and 2 conventional splenic DCs from WT, p84 and p101-deficient mice 10 days after Flt3L-B16 melanoma injection. The DCs were enriched by anti-CD11c magnetic beads before the cell sorting. MHC-II^hi^, CD11c^hi^ and XCR1^+^ cells were sorted as type 1 DCs, while MHC-II^hi^, CD11c^hi^ and Sirpα^+^ were sorted as type 2 DCs.

**(B)** The graphs show data from 4 cell sorting experiments performed as in (A) (ns: non-significant; *: 0.01<p<0.05, one-way ANOVA test followed by Dunnett’s multiple comparisons).


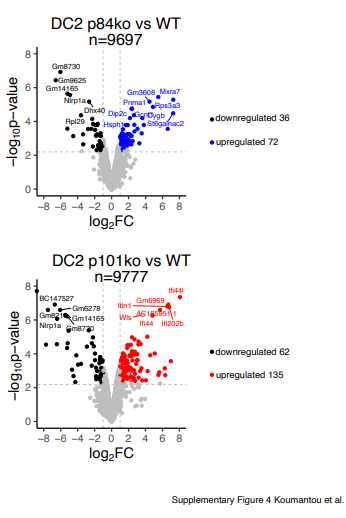


**Supplementary Figure 4. The deletion of p84 or p101 regulatory subunits does not cause a major shift in the transcriptomic profile of type 2 splenic DCs**.

Volcano plots showing the significantly upregulated and downregulated genes in p84ko (upper graph) and p101ko (lower graph) type 2 DCs compared with the WT cells. The names of the 15 most significantly up- or down-regulated genes are shown in the plots. “n” represents the number of transcripts for each plot.


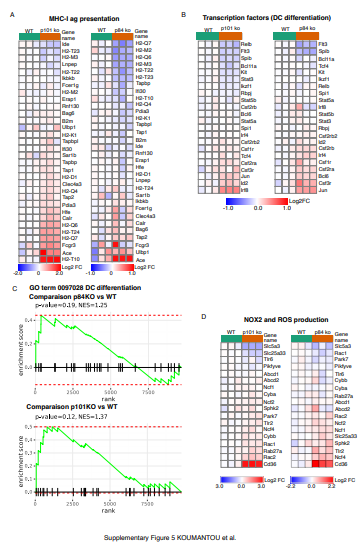


**Supplementary Figure 5. The transcription of genes coding for antigen processing and presentation machinery, NOX2 complex members and DC differentiation-related transcription factors, is not affected by p84 or p101 deletion.**

**(A)** Heat maps showing the differential expression of identified genes after RNA sequencing that belong to the GO term 0002474 (log2 FC).

**(B)** Heat maps showing the differential expression of identified genes encoding transcription factors implicated in dendritic cell differentiation in murine splenic type 2 DCs, as assessed by bulk RNA sequencing (log2 FC).

**(C)** Graphical representation of the gene set enrichment analysis using the biological process GO term 0097028 (dendritic cell differentiation) in p84ko versus WT (top) and p101ko versus WT (bottom).

**(D)** Heat maps showing the differential expression of identified genes after RNA sequencing that belong to the GO terms 0043020 and 1903426 (log2 FC).


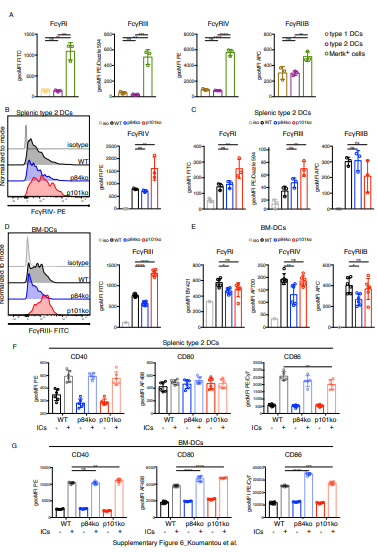


**Supplementary Figure 6. Surface expression of FcγRs and co-stimulatory molecules in splenic conventional DCs and GM-CSF-derived iDCs.**

**(A)** The cell surface expression of the α chain of FcγRs in splenic type 1 and type 2 DCs was assessed by flow cytometry. Mertk^+^ macrophages cells were used as a positive control of FcγRs expression. Each dot represents an individual mouse (ns: non-significant; *: 0.01<p<0.05; **0.001<p<0.01; **0.001<p<0.01; ****p<0.0001, unpaired Student t test)

**(B)** Histograms and bar graph showing the surface expression levels of the activating FcγRIV in wt, p84 and p101-deficient splenic type 2 DCs. Each dot represents an individual mouse (ns: non-significant; **0.001<p<0.01, one-way ANOVA test followed by Dunnett’s multiple comparisons).

**(C)** Graphs showing the surface expression intensity of FcγRI, FcγRIII and FcγRIIB in wt, p84 and p101-deficient splenic type 2 DCs. Each dot represents an individual mouse (ns: non-significant; **0.001<p<0.01, one-way ANOVA test followed by Dunnett’s multiple comparisons).

**(D)** Histograms and bar graph showing the surface expression levels of the activating FcγRIII in wt, p84 and p101-deficient GM-CSF-derived iDCs. The experiment was performed twice in triplicates (****: p<0.0001, one-way ANOVA test followed by Dunnett’s multiple comparisons).

**(E)** Graphs showing the surface expression intensity of FcγRI, FcγRIV and FcγRIIB in wt, p84 and p101-deficient GM-CSF-derived iDCs. The experiment was performed twice in triplicates (ns: non-significant; *: 0.01<p<0.05; **0.001<p<0.01, one-way ANOVA test followed by Dunnett’s multiple comparisons).

**(F)** Graphs showing the plasma membrane expression of the co-stimulatory molecules CD40, CD80 and CD86 in splenic wt, p84ko and p101ko type 2 DCs at steady state and after intraperitoneal injection of ICs. The data are derived from two individual experiments. Each dot represents an individual mouse (**0.001<p<0.01, one-way ANOVA test followed by Dunnett’s multiple comparisons).

**(G)** Graphs showing the plasma membrane expression of the co-stimulatory molecules CD40, CD80 and CD86 in wt, p84 and p101-deficient GM-CSF-derived iDCs at steady state and after 24h incubation with ICs. The experiment was performed twice in triplicates (ns: non-significant; **0.001<p<0.01; **0.001<p<0.01; ****p<0.0001, one-way ANOVA test followed by Dunnett’s multiple comparisons).


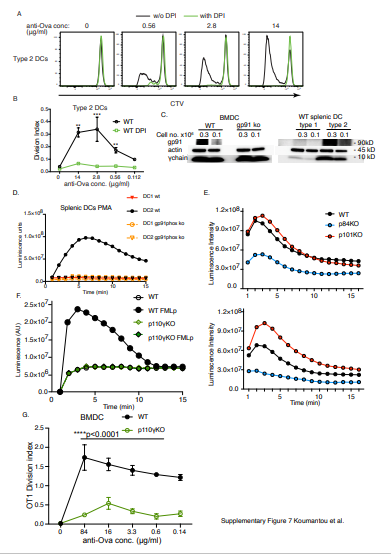


**Supplementary figure 7. ROS production is required for cross-presentation by type 2 DCs**

(**A**) WT type 2 splenic DCs were incubated for 16h with Ova-antiOva ICs, without or with 1mM DPI (Diphenyleneiodonium), a general NOX inhibitor. After washing, the DCs were incubated with CTV-labeled naïve OT1 T cells. The OT1 T cell activation was measured by CTV dye dilution.

**(B)** The graph shows the division index of activated T cells. Data are representative of 2 independent experiments realized in duplicate (**p<0.01; ***p<0.001, two-way ANOVA followed by Sidak’s multiple comparisons).

**(C)** Expression of gp91phox subunit of NOX2 complex and of FcRs γ-chain in WT and gp91phox-deficient conventional and GM-CSF-derived iDCs was analyzed by immunoblot. Actin was used as a loading control. Related to the Figure 4B.

**(D)** Type 1 and 2 splenic conventional DCs were isolated by cell sorting from WT and gp91phox-deficient mice and their ability to produce ROS after PMA stimulation was measured by luminol-amplified chemiluminescence. Related to the Figure 4C.

**(E)** WT, p84 and p101-deficient GM-CSF-derived iDCs at day 7 were tested for their ability to produce ROS after the activation of Formyl peptide receptor (FPR) with N-Formylmethionyl-leucyl-phenylalanine (fMLP), as in (C). Related to the Figure 4D.

**(F)** WT and p110γ-deficient GM-CSF-derived iDCs (BMDC) were differentiated *in vitro* with GM-CSF for 7 days. Their ability to produce ROS after the activation of Formyl peptide receptor (FPR) with N-Formylmethionyl-leucyl-phenylalanine (FMLp) was measured by luminol-amplified chemiluminescence. Related to the Figure 5A.

(**G**) WT and p110γ-deficient GM-CSF-derived iDCs incubated for 16h with Ova anti-Ova immune complexes were used to stimulate naïve OT1 T cells. The OT1 T cell activation was measured by CTV dye dilution. The graph shows the division index of activated T cells. Related to the Figure 5C (p<0.0001, two-way ANOVA followed by Sidak’s multiple comparisons).
